# Survival outcomes are associated with genomic instability in luminal breast cancers

**DOI:** 10.1371/journal.pone.0245042

**Published:** 2021-02-03

**Authors:** Lydia King, Andrew Flaus, Emma Holian, Aaron Golden

**Affiliations:** 1 The SFI Centre for Research Training in Genomics Data Sciences, National University of Ireland Galway, Galway, Republic of Ireland; 2 Bioinformatics and Biostatistics Research Cluster, School of Mathematics, Statistics and Applied Mathematics, National University of Ireland Galway, Galway, Republic of Ireland; 3 Centre for Chromosome Biology, Biochemistry, School of Natural Sciences, National University of Ireland Galway, Galway, Republic of Ireland; Universitat Zurich, SWITZERLAND

## Abstract

Breast cancer is the leading cause of cancer related death among women. Breast cancers are generally diagnosed and treated based on clinical and histopathological features, along with subtype classification determined by the Prosigna Breast Cancer Prognostic Gene Signature Assay (also known as PAM50). Currently the copy number alteration (CNA) landscape of the tumour is not considered. We set out to examine the role of genomic instability (GI) in breast cancer survival since CNAs reflect GI and correlate with survival in other cancers. We focused on the 70% of breast cancers classified as luminal and carried out a comprehensive survival and association analysis using Molecular Taxonomy of Breast Cancer International Consortium (METABRIC) data to determine whether CNA Score Quartiles derived from absolute CNA counts are associated with survival. Analysis revealed that patients diagnosed with luminal A breast cancer have a CNA landscape associated with disease specific survival, suggesting that CNA Score can provide a statistically robust prognostic factor. Furthermore, stratification of patients into subtypes based on gene expression has shown that luminal A and B cases overlap, and it is in this region we largely observe luminal A cases with reduced survival outlook. Therefore, luminal A breast cancer patients with quantitatively elevated CNA counts may benefit from more aggressive therapy. This demonstrates how individual genomic landscapes can facilitate personalisation of therapeutic interventions to optimise survival outcomes.

## Introduction

Breast cancer is one of the most common malignancies affecting women worldwide and is the leading cause of cancer related death among this group [1, 2]. Over 2 million new breast cancer cases were reported in 2018 and it is estimated that over 40,000 people will die as a result of breast cancer in the United States in 2019 [1, 3].

Breast cancer was previously treated as a single disease, but recent advances in areas such as next generation sequencing have led to it being regarded as a collection of highly heterogeneous diseases with distinct molecular and clinical phenotypes including disease progression rate, treatment response and survival [4–6]. Despite these advances, certain aspects of breast cancer treatment still apply standards of care to broad patient cohorts. For example, as no valid predictive factors for radiotherapy response have yet been identified, it is recommended that radiotherapy should be considered for all patients undergoing breast conserving surgery irrespective of whether their tumours are likely to respond or not, with the decision often being determined on the basis of clinical stage and standard histopathological criteria [7].

The molecular classification of breast cancer currently makes use of PAM50 intrinsic subtyping determined by the Prosigna Breast Cancer Prognostic Gene Signature Assay (formerly called the PAM50 test) based on gene expression profiling [8–10]. This distinguishes luminal A (lumA), luminal B (lumB), human epidermal growth factor receptor 2 (*HER2*)-enriched and basal-like subtypes [8]. The differences in gene expression patterns among these intrinsic subtypes reflect basic alterations in the cell biology of the tumours [11]. Importantly, it has been observed that ∼ 85% of the variations in gene expression patterns of breast cancers are as a result of copy number alterations (CNAs) [5, 12].

Approximately 70% of breast cancers belong to the luminal subtypes lumA and lumB characterised by increased levels of estrogen receptor (ER) and progesterone receptor (PR) [13]. LumA tumours display lower levels of genomic instability (GI) compared to lumB tumours [11]. GI is regarded as a hallmark of cancer and refers to an increased tendency toward alterations in the genome during the life of cells [14]. These alterations range from single nucleotide changes to large scale structural reorganisation of chromosomes, aneuploidy and whole genome duplications [14]. GI can initiate cancer, affect progression and influence patient prognosis [15].

Recent studies suggest that the relationship between lumA and lumB may be a continuum rather than a strict division of subtypes [11–13]. It has also been hypothesised that lumA tumours may evolve into lumB tumours as a result of stochastic acquisitions of mutations in genes associated with worse prognosis, including *HER2* and tumour protein p53 (*TP53*) [13, 16].

At present, breast cancer diagnosis and treatment follows an integrative approach whereby both clinical and histopathological features such as age at diagnosis, tumour size, lymph node metastasis and histological grade are utilised alongside tissue derived biomarkers [17]. However, it is widely accepted that breast cancer is largely dominated by chromosomal rearrangements [5, 18, 19], and a growing body of evidence suggests that the incorporation of the genomic landscape of the tumour into treatment decisions is extremely beneficial to the patient [20, 21].

Several studies have shown that the copy number landscape of a tumour can affect survival [5, 22, 23]. A pan-cancer analysis suggests that the CNA burden measured as the percentage of the tumour genome with CNAs is associated with both overall survival (OS) and disease specific survival (DSS) in a range of cancers including breast, endometrial, renal, thyroid, and colorectal cancer [23]. Assessing aneuploidy in prostate cancers at diagnosis has been shown to be more predictive of long term survival than the Gleason score which is the standard clinical metric [22]. Consistent with this, Zhang et al [5] reported an association between general CNA burden and breast cancer survival in the Molecular Taxonomy of Breast Cancer International Consortium (METABRIC) dataset.

These studies support the conjecture that the CNA landscape of a tumour is itself associated with both OS and DSS, and could provide a prognostic biomarker [5, 22, 23]. The original association in breast cancer reported by Zhang et al [5] considered all PAM50 intrinsic subtypes and used a simple binary measure of CNA burden to categorise GI. Earlier, Tishchenko et al [13] used a continuous cytoband-based measure of CNA gain or loss rate correlated to local gene expression to quantify CNA variation.

We hypothesised that a more nuanced measure of CNA burden could provide additional prognostic information for the impact of GI on luminal breast cancer survival, and potentially be informative for the progression from lumA to lumB subtypes.

## Materials and methods

**Fig 1:**
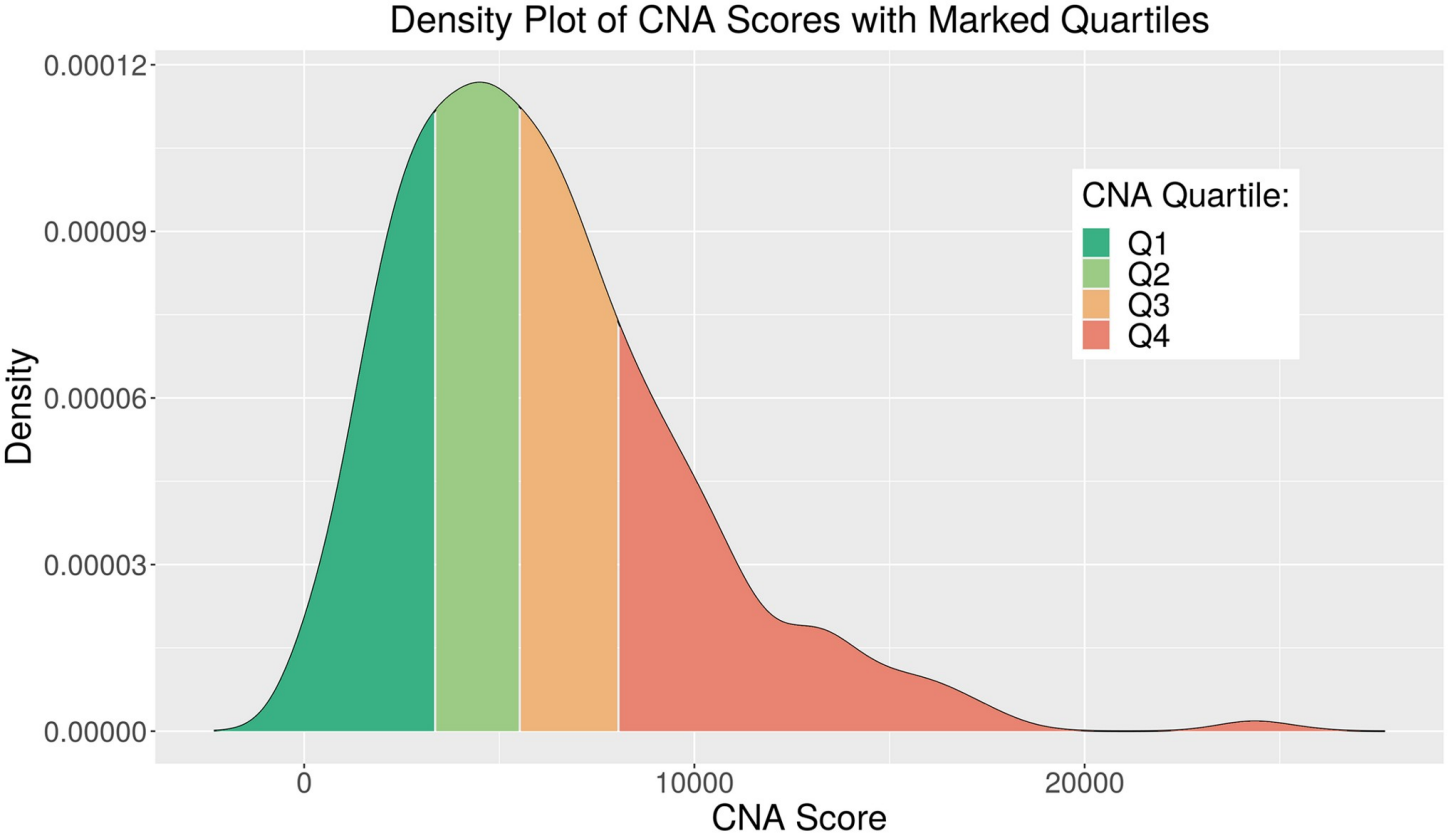
Density plot of CNA Score distribution for METABRIC luminal cases. CNA Quartiles 1-4 with threshold scores ∼3335, ∼5547 and ∼8064 respectively, indicated by legend colours.

A CNA Score metric was developed using the absolute CNA profiles of all luminal patients profiled within the METABRIC consortium. This was calculated by summing the absolute value of the scores for all genes. Cases were then assigned to ranked quartiles as a first-order means of segmentation for analysis (Fig 1 and S1 Table in S1 File).

Survival analysis was carried out for these CNA Quartiles using associated clinical data to determine survival associated variables. Statistical association tests were then applied to validate that the association between a given CNA Quartile and its survival outcome was due to the CNA Quartile and not to a confounding variable. Finally, multivariable Cox proportional hazards (PH) models and the associated assumption tests were used to confirm that the survival outcomes are associated with GI in specific cohorts of luminal breast cancers and survival tree recursive partitioning was used to further explore survival interactions and cut-off points. In addition, quantile classification from the gene expression analysis of Tishchenko et al [13] was utilised to examine the luminal stratification associated with cases where GI affects survival. Going forward, to adequately distinguish our CNA Quartiles from the gene expression quantile classification of Tishchenko et al [13], our CNA Quartiles are referenced using a capital Q while the Tishchenko quantiles are referenced using a lower-case q. All analysis was conducted using the R statistical processing environment, and an R Shiny app was subsequently developed to expedite this work (manuscript in preparation).

### METABRIC data

METABRIC provides a well-annotated dataset of over 2,000 breast cancer cases with long-term clinical follow-up data, transcriptomic and genomic data [12]. Luminal cases have an average follow-up time of 130.5 months (10.9 years) months and a maximum follow-up time of 337 months (28.1 years). All CNA profiles, clinical patient and sample annotations for luminal patients (n = 1175) were obtained from cBioPortal [24]. The METABRIC consortium [12] utilised both the circular binary segmentation algorithm [25] and an adapted Hidden Markov model [26] for segmentation, followed by CNA calling. The patient-specific somatic CNA profile calls for each gene have values indicating homozygous deletion (-2), hemizygous deletion (-1), diploidy (0), single copy gain (+1) and high level amplification (+2). Quantile classification based on relative gene expression for luminal METABRIC cases was obtained from Tishchenko et al [13].

### Statistical analyses

**Fig 2:**
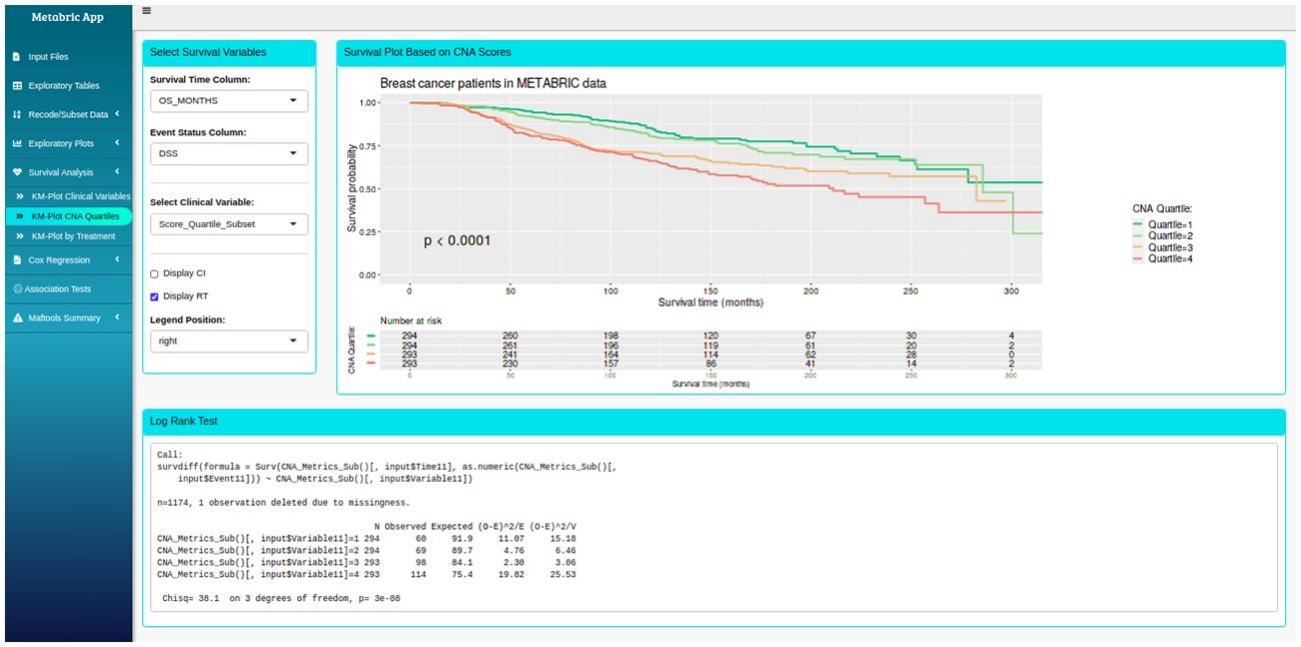
Graphical user interface (GUI) of the R shiny app (manuscript in preparation). Displayed here is the Survival Analysis tab showing the DSS based on CNA Quartiles in luminal cases. Results from the corresponding log-rank test are displayed in the bottom box.

Clinical data and CNA profiles were formatted and analysed using R (version 3.5.1) and RStudio (version 1.2.1335) with R packages *survival*, *survminer* and *gglplot2* [27–29]. Additional functions such as mutation analysis using the R package *maftools* [30] were also implemented. These packages and associated processing scripts were packaged into a bespoke R Shiny app with multiple tab panels capable of running and displaying the results of the entire statistical analyses (Fig 2). Sidebar tabs include Input Files, Exploratory Tables, Recode/Subset Data, Exploratory Plots, Survival Analysis, Cox Regression, Association Tests and Maftools Summary. The app provided a rapid, powerful and effective means to explore, segment, visualise and statistically test the METABRIC data. Survival analysis using Kaplan-Meier (KM) plots and univariate Cox models were applied to OS and DSS outcomes with CNA Quartiles and with each clinical variable. A multivariable Cox PH model was fitted to OS and DSS outcome with CNA Quartiles and selected candidate clinical variables. DSS outcome was also analysed by survival tree recursive partitioning using the R package *partykit* [31, 32] with CNA Score and candidate clinical predictors to explore interactions and determine optimised cut-off points in CNA Score.

## Results

### Survival outcome is associated with CNA Quartile

#### Univariate survival analysis of luminal breast cancers with CNA Quartiles

**Fig 3:**
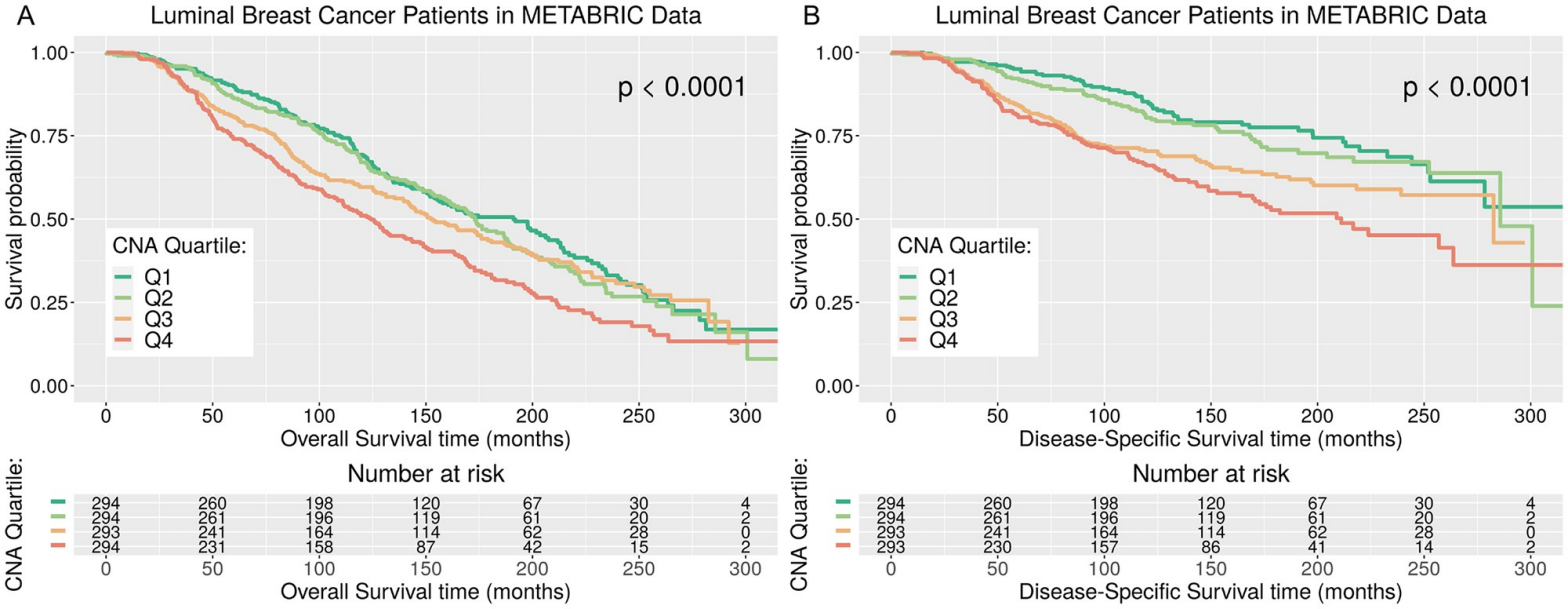
KM plots for OS and DSS for METABRIC luminal breast cancer patients in each CNA Quartile. Kaplan Meier plots and risk tables for luminal breast cancer survival. Overall Survival (OS), Fig (A), and Disease Specific Survival (DSS), Fig (B), for METABRIC luminal breast cancer patients in each CNA Quartile. The *p*-value associated with the log-rank test and a risk table displaying the number of patients at risk at each time interval is displayed.

A number of recent studies report that CNAs reflecting GI are associated with survival outcomes in several types of cancer [5, 22, 23]. We hypothesised that CNA Quartiles based on absolute CNA Score would be associated with both OS and DSS in luminal breast cancer patients. Separate KM survival curves for patients in the four CNA Quartiles for OS outcome and DSS outcome (Fig 3) show patients in CNA Quartile 4 (Q4), with higher CNA Score values indicative of higher levels of GI, have worse survival outcomes than patients with less GI in CNA Quartiles 1-3 (Q1-3). The median OS time for patients in the category with highest GI, CNA Q4, is observed to be 124.2 months, a reduction compared to median OS time observed for patients in CNA Q3, Q2 and Q1, which are 152.33, 173.03 and 191 months respectively, and the differences in the four OS curves are deemed significant using the log-rank test (p-value < 0.0001). The median DSS time for patients in the category with highest GI, CNA Q4, is observed to be 211.13 months, a reduction compared to median DSS time observed for patients in CNA Q3, Q2 and Q1, which are 282.57 and 285.7 months, for Q3 and Q2, while the median survival time for CNA Q1 cannot be determined as it not within the range observed, i.e. > 300 months. Differences in the four DSS survival curves are deemed significant using the log-rank test (p-value < 0.0001).

#### Analysis of possible confounding variables and multivariable Cox models

A strong association between breast cancer survival and clinical variables such as PAM50 subtype, clinical stage and age at diagnosis has been reported [33–35]. A number of steps were taken to determine whether the association between survival outcomes and CNA Quartiles was the result of confounding variables, which are additional factors influencing survival outcomes that are also correlated with CNA Quartiles.

Survival analysis using KM plots and univariate Cox models were carried out to identify whether any of 23 clinical variables (S2 Table in S1 File) available for the luminal data were associated with survival outcome. We found that 19 of the clinical variables were associated with OS and 18 were associated with DSS (S3, S4 Tables in S1 File). These were taken forward for examination using statistical tests. A *χ*^2^ test was used to test the association between two categorical variables with sufficient cell sizes in the two-way table of categorical variables. Fisher’s exact test was used in the case where any cell size was sufficiently small. The non-parametric Kruskal-Wallis test was used to determine if there were statistically significant differences between CNA Quartiles and continuous clinical variables associated with survival outcomes. These tests indicated that the CNA Quartiles are significantly associated with a number of clinical variables (S5 Table in S1 File).

**Fig 4:**
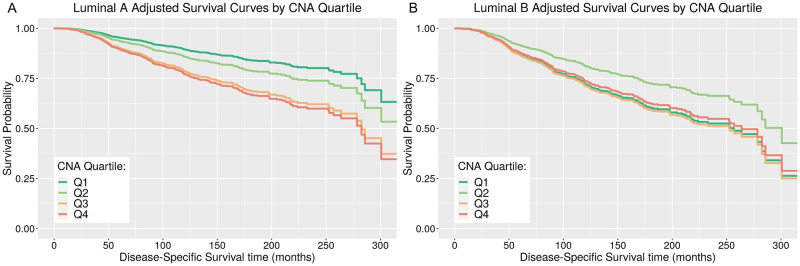
Adjusted survival curves for estimated CNA Quartile effects within each luminal PAM50 subtype. Adjusted survival curves for estimated CNA Quartile effects for METABRIC luminal A, Fig (A) and luminal B, Fig (B) breast cancer patients. Survival curves represent the estimated effect of CNA Quartiles, by plotting the predicted survival curves for luminal A and luminal B patients for each CNA Quartile, having adjusted for the effects of other covariates in the multivariable Cox model, where other covariates are fixed at the median/mode values of those variables.

Following clinical variable selection for outcome DSS, eight candidate clinical predictors (Section 5 and S6–S9 Tables in S1 File) and the CNA Quartile variable were fitted to model OS and DSS outcome using multivariable Cox PH models, and the effect of CNA Quartiles on survival was examined. Assuming PH, the results indicated that CNA Quartiles are significantly associated with DSS along with 6 of the clinical predictors: PAM50 subtype, histological grade, tumour size, number of positive lymph nodes, age at diagnosis, and HER2 status (Table 1). The reference group in the model is lumA, histological grade 1, HER2-negative patients with CNA Scores in CNA Q1. The quantitative predictors in this model, tumour size, positive lymph nodes and age at diagnosis, show a significant increased risk in DSS with each unit increase in the predictor with estimated hazard ratios, 1.015, 1,051, 1.018, respectively (p-values < 0.001). Comparing histological grade 3 to grade 1 shows a significant increased risk in DSS, with estimated hazard ratio 1.696 (p-value 0.044). There was no evidence of a significant effect on risk comparing histological grade 2 to grade 1 (p-value 0.134). Comparing HER2-positive patients to HER2-negative patients gives a significant increased risk in DSS, with an estimated hazard ratio 1.717 (p-value 0.007). Comparing lumB patients to lumA patients gives a significant increased risk in DSS, with estimated hazard ratio 2.912 (p-value < 0.001). For lumA patients, comparing CNA Q4 patients to CNA Q1 patients, i.e. highest categorisation of GI to lowest categorisation of GI, shows a significant increased risk in DSS, with estimated hazard ratio 2.315 (p-value 0.002). Comparing CNA Q3 patients to CNA Q1 patients shows a significant increased risk in DSS, with estimated hazard ratio 2.152 (p-value 0.002). There was no evidence of a significant effect on risk for lumA patients comparing CNA Q2 patients to CNA Q1 patients (p-value 0.219). For lumB patients, the effect of CNA Quartile on DSS differs in comparison to effects estimated for lumA patients, estimated by fitting interaction effects between CNA Quartiles and PAM50 subtype, where the effect is a reduction in the estimated CNA Quartile differences for lumB, estimated coefficients -0.764, -0.73, and 0.909 (p-values 0.053, 0.045 and 0.014) for CNA Q2, Q3, Q4 respectively. Fig 4 illustrates the survival curves, as estimated from this model adjusting for covariates, specifically for lumA patients within the different CNA Quartiles (Fig 4A) and for lumB patients within the different CNA Quartiles (Fig 4B). Differences observed in DSS curves comparing CNA Quartiles within lumB are small and can be shown to be not significant by reparametrizing the model reference group to be lumB (S12 Table in S1 File).

**Table 1:** Final multivariable Cox PH model with selected clinical variables, CNA Quartiles and interactions.

Clinical Variable	Beta	SE	HR	95% CI	P-value	Significance
PAM50:						
Luminal A (Ref)	-	-	-	-	-	-
Luminal B	1.069	0.299	2.912	(1.619—5.237)	<0.001	***
Histological Grade:						
1 (Ref)	-	-	-	-	-	-
2	0.381	0.254	1.464	(0.889—2.410)	0.134	
3	0.528	0.262	1.696	(1.014—2.837)	0.044	*
Tumour Size	0.015	0.003	1.015	(1.010—1.020)	<0.001	***
Positive Lymph Nodes	0.050	0.008	1.051	(1.034—1.069)	<0.001	***
Age at Diagnosis	0.018	0.005	1.018	(1.008—1.029)	<0.001	***
HER2 Status:						
Negative (Ref)	-	-	-	-	-	-
Positive	0.541	0.202	1.717	(1.157—2.550)	0.007	**
CNA Quartile:						
CNA Q1 (Ref)	-	-	-	-	-	-
CNA Q2	0.315	0.256	1.370	(0.829—2.265)	0.219	
CNA Q3	0.767	0.247	2.152	(1.326—3.493)	0.002	**
CNA Q4	0.839	0.272	2.315	(1.360—3.942)	0.002	**
CNA Quartile by PAM50:						
CNA Q2:LumB	-0.764	0.395	0.466	(0.215—1.010)	0.053	.
CNA Q3:LumB	-0.730	0.364	0.482	(0.236—0.983)	0.045	*
CNA Q4:LumB	-0.909	0.370	0.403	(0.195—0.831)	0.014	*
Likelihood Ratio Test p-value					<2e-16	***
Wald Test p-value					<2e-16	***
Score (logrank) Test p-value					<2e-16	***

Signifcance codes: 0(***) 0.001(**) 0.01(*) 0.05(.) 0.1()

SE: Standard Error; HR: Hazard Ratio; CI: Confidence Interval

#### Recursive partitioning survival trees

**Fig 5:**
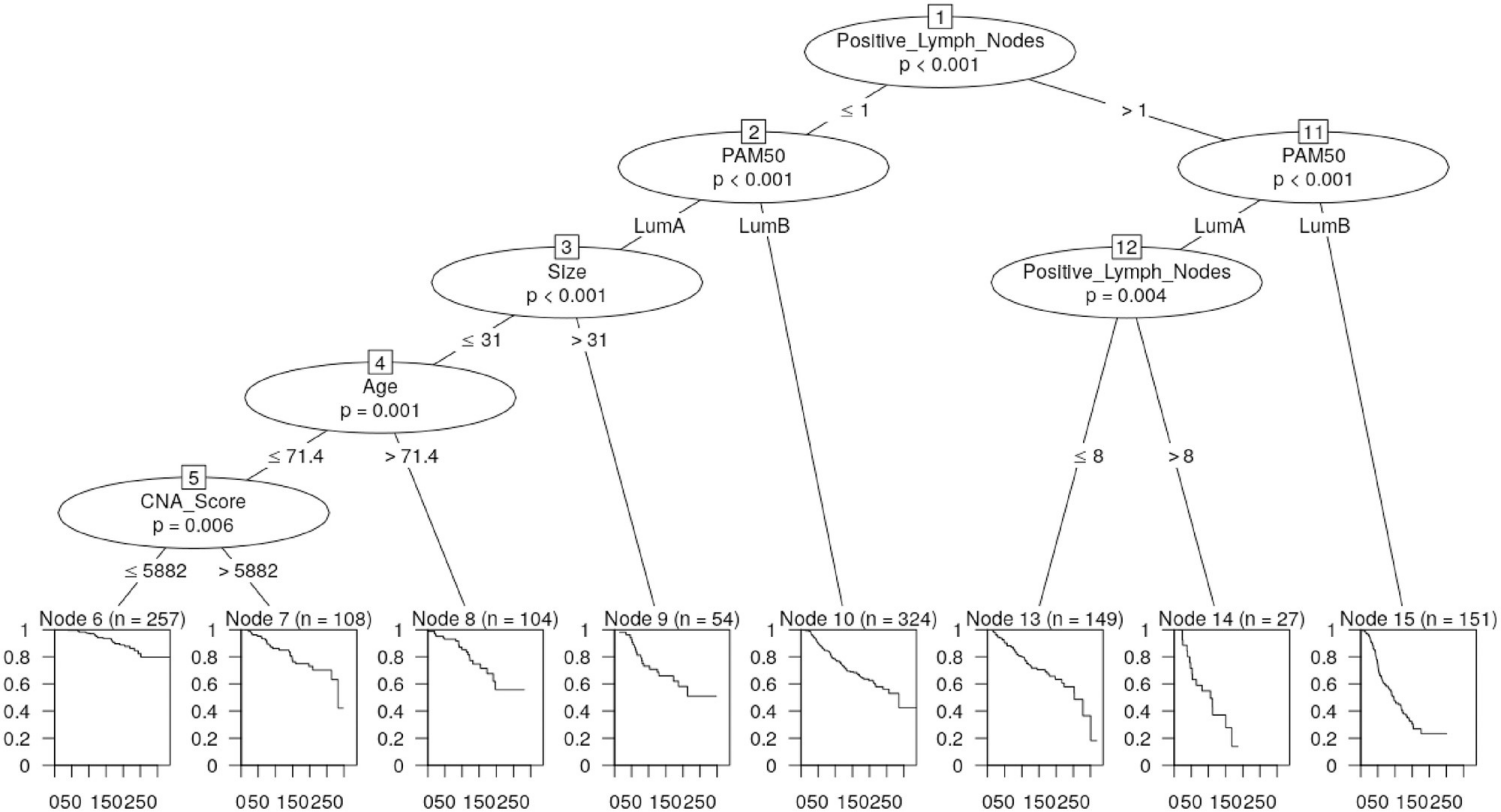
Recursive partitioning survival tree utilising the six significant clinical variables and CNA Score.

Diagnostic tests indicated that the PH assumption may not be met, although this can usually be addressed by fitting a Cox model stratified across values of the variable if the variable is not the main effect of interest. CNA Quartile is the main effect of interest in this application so we proceeded to apply recursive partitioning survival trees. In addition, survival trees help to examine any interactions between the six significant clinical variables and CNA Score in modelling DSS, and to determine the optimum cut-off in CNA Score (Fig 5 and S1 Table in S1 File).

This confirmed that CNA Score is a significant predictor for DSS outcome along with the number of positive lymph nodes, PAM50 subtype, tumour size, and age of diagnosis.

For lumA patients who have 0-1 positive lymph nodes, tumour size less than 31mm and age of diagnosis less than 71.4 years, the outcome DSS can be stratified by CNA Score with optimised CNA Score cut-off point value 5882 (Fig 5, p-value 0.006). Patients with CNA Score above 5882 showed reduced survival probability than a CNA Score representing lower GI. Interestingly, this cut-off is close to the discrimination of CNA Q2 and Q3 at a CNA Score of ∼ 5547 (S1 Table in S1 File).

Survival trees considering CNA Quartiles instead of the raw CNA Score suggested a similar partitioning with CNA Q1 and Q2 versus CNA Q3 and Q4 (S1 Fig in S1 File). This illustrates that CNA Quartile can segregate lumA patients in predicting DSS, consistent with the effects estimated by the Cox PH model.

Overall, the survival trees indicate that the CNA Score metric implemented either as a CNA Quartile or a raw value can stratify subsets of patients based on DSS, and therefore illustrates that CNA Score can identify lumA patients who are at elevated risk.

### Stratification of luminal cancers

Analyses carried out by Tishchenko et al [13] on the transcriptomic and genomic landscape of luminal breast cancers in both the METABRIC and Research Online Cancer Knowledgebase (ROCK) datasets suggested that the rigid stratification of luminal breast cancers into lumA and lumB intrinsic molecular subtypes is equivocal. These authors identified the top ten most up-regulated genes in all luminal samples and observed that they were primarily associated with cell proliferation (S14 Table in S1 File).

Four quantiles (Tishchenko quantiles) comprising approximately a quarter of METABRIC luminal patients each were defined by Tishchenko et al [13] based on the relative expression of the top ten up-regulated genes. The progression from Tishchenko quantile 1 to 4 (q1-4) showed an increase in patient risk level and an approximately continuous transition in the proportion of lumA to lumB subtypes. The mixing between lumA and lumB classification begins in Tishchenko q2 and reaches a peak of ambiguity in Tishchenko q3 with some mixing still observed in Tishchenko q4. The authors proposed that this reflects a continuous variation of a molecular profile with increasing genomic damage [13].

**Fig 6:**
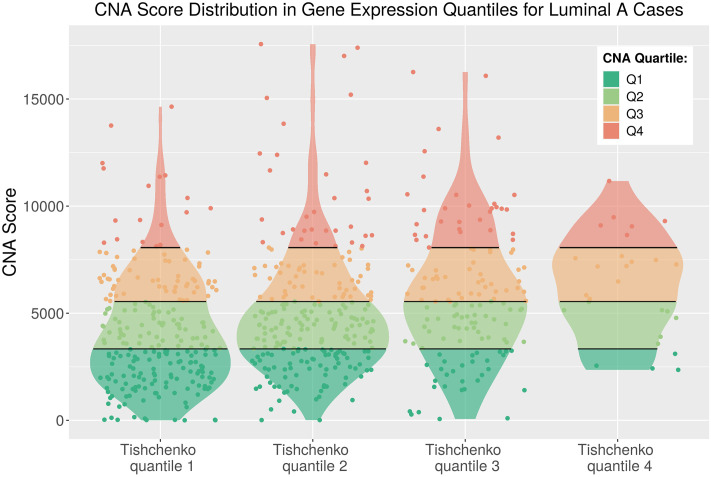
CNA Score distribution in gene expression quantiles of Tishchenko et al [13] for ordinary luminal A cases. CNA Quartile thresholds from Fig 1 indicated across violin area. Outliers were removed.

Our CNA Score is associated with the transcriptomic ranking of Tishchenko et al [13]. Cross referencing revealed that 666 lumA cases were common to this study and the Tishchenko et al [13] ordinary luminal set. A noticeable overlap was observed between patients sharing both a CNA Score in CNA Q3-4 associated with poor DSS outcome from our work and a transcriptome signature associated with a similarly poor outlook from the study of Tishchenko et al [13] (Fig 6 and S2 Fig in S1 File). A large proportion of the lumA CNA Q3 and Q4 cases have gene expression levels in the transition between lumA and lumB subtype classification assigned by Tishchenko et al [13] to Tishchenko q2, q3 or q4 (Table 2). Together with the association of CNA Score and survival outcome for lumA cases, this suggests GI profiles could be informative for treatment decisions for luminal breast cancers.

**Table 2:** Two-way table of patient counts in CNA Quartiles and gene expression quantiles of Tishchenko et al [13] for ordinary luminal A cases.

	**CNA Q1**	**CNA Q2**	**CNA Q3**	**CNA Q4**	**Total**
**Tishchenko q1**	111	62	56	19	248
**Tishchenko q2**	76	88	50	34	248
**Tishchenko q3**	30	40	41	32	143
**Tishchenko q4**	4	7	10	6	27
**Total**	221	197	157	91	666

## Discussion

We analysed the effect of CNA burden on breast cancer survival by implementing a series of statistically robust tools for interrogation of the rich and well annotated METABRIC dataset. We assigned each patient to a group by quartile segmentation of the summed distribution profile of absolute CNA Score values, providing a first order measure of CNA burden. This metric is a more nuanced representation of GI compared to the binary segmentation used in previous studies [5]. Our analysis revealed that the presence of high CNA levels in the tumour genomes of patients with lumA breast cancer is associated with worse DSS outcomes, based on the clinical information and CNA profiles of 1175 luminal breast cancer patients registered in the METABRIC archive.

The observed difference in survival outcomes could be the result of either a true association between survival and CNA Quartiles, or the result of confounding factors. A significant association was again observed between survival outcomes and CNA Quartiles for lumA patients utilising multivariable Cox PH models to address potential confounders. The association with DSS was linear in lumA patients where DSS outcomes decreased moving from CNA Q1 to Q4. Overall, CNA Quartiles were associated with breast cancer prognosis independent of other strong clinical predictors.

Recent studies have proposed that lumA tumours may evolve into lumB tumours through the stochastic acquisitions of mutations in genes associated with worse prognosis [13, 16]. Tishchenko et al [13] used the top ten up-regulated genes in all METABRIC luminal cases to rank and assign quantiles with approximately 25% of patients in each group. The incidence of lumB tumours was found to increase along with patient risk level in the progression from Tishchenko q1 to q4. This led the authors to hypothesise that luminal tumours represent a continuum whose subtype range correlates with increasing genomic damage.

The lumA tumours identified in our analysis as associated with worse DSS outcomes largely correspond to Tishchenko q2, q3 and q4 of the Tishchenko et al study [13]. This means they occupy the region of PAM50 subtype stratification where the boundaries between lumA and lumB cases overlap. Therefore, our work provides further support for the proposal of a gradient in luminal classification by providing a robust statistical validation of the association between CNA burden and survival outcome for lumA cases at the boundary where lumA and lumB cases overlap in cell proliferation gene expression.

LumA patients who belong to both higher CNA Quartiles and higher gene expression Tishchenko quantiles are at particular risk for long term survival outcome. This has potential clinical utility because these patients are potentially not well stratified by the PAM50 subtype but can be identified by the CNA Score. Therefore, patients classified as lumA that display gene expression levels more akin to lumB tumours and also have high CNA burden may benefit from the more aggressive treatment regime used for lumB patients in contrast to standard endocrine therapy for lumA patients [36].

We defined the CNA Score metric as the sum of the absolute CNA values over all genes per patient. This definition enables unbiased analysis, maintains the easy interpretation of the data, and provides sufficient samples per CNA Quartile to implement meaningful statistical analyses. However, this definition is a simplification of the CNA landscape in tumour cells. Fine grained features including length of the CNA, whether it is an amplification or deletion, and the genomic location of the CNA are not considered. The analysis could potentially be made more sensitive with a richer metric, although the smaller sample groups available following such fine grained segmentation of the METABRIC luminal cohort would likely compromise rigorous statistical analysis. To avoid sample size limitations, expanded datasets that combine high quality genomic and transcriptomic profiling with long-term clinical follow-up are required to provide sufficient cases for independent discovery and validation.

Overall, this work demonstrates a practical pathway towards personalised medicine using genomic characteristics. Such an individualised approach to classifying breast cancers could improve the success of treatment interventions by guiding tailored therapeutic strategies based on the genomic profile of an individual tumour [37]. For example, a simple measure of CNA burden obtained from biopsy or resected tumour sample material could provide a prognostic biomarker to stratify a luminal breast cancer patient in addition to histological grade and PAM50 subtype.

## Conclusion

It is important to identify features of luminal breast cancer that have statistically robust prognostic value in order to identify patients with a greater risk of lethal disease because the number of women diagnosed is increasing and the majority of cases belong to luminal subtypes. We analysed freely available clinical and genomic patient data from the METABRIC dataset to study the impact of CNA burden on overall survival within the luminal subtypes. We observed that CNA Quartiles based on the sum of absolute CNA variations are a prognostic factor for breast cancer survival outcomes in a subset of patients with high GI suffering from lumA breast cancer. We further demonstrated that some of the lumA cases in our study lie in the ambiguous region between lumA and lumB subtype classifications identified in an earlier analysis of gene expression levels from the same METABRIC patient samples. Women diagnosed with lumA breast cancer who possess a CNA burden within our derived CNA Q3 or Q4 have reduced survival outcomes and may benefit from more aggressive therapy. This progresses efforts to incorporate individual genomic landscapes into more nuanced classifications of breast cancer cases, with the goal of personalising therapeutic interventions to optimise long term survival outcomes for patients.

## Supporting information

S1 File (PDF)
