# Supplementary material for: Survival outcomes are associated with genomic instability in luminal breast cancers
Source: PLoS One. 2021 Feb 3;16(2):e0245042. doi: 10.1371/journal.pone.0245042 (PMC7857737; doi:10.1371/journal.pone.0245042)
Supplement: S1 File — (PDF) [file pone.0245042.s001.pdf]

Survival Outcomes are Associated with Genomic Instability in Luminal Breast Cancers: Supplementary Material

Lydia King, Andrew Flaus, Emma Holian and Aaron Golden.

Contents

1 CNA Quartile Cut-Offs **2**

2 Description of Clinical Characteristics **3**

3 Univariate Cox Models **4**
3.1 Overall Survival . . . 4
3.2 Disease-Specific Survival . . . 5

4 Association Analysis **6**

5 Clinical Variable Selection and Summaries **7**
5.1 Clinical Variable Selection . . . 7
5.2 Selected Clinical Variable Summaries . . . 8
5.2.1 All Luminal Cases . . . 8
5.2.2 Luminal A Cases . . . 9
5.2.3 Luminal B Cases . . . 9
5.2.4 Luminal A Histological Grade 3 Cases . . . 10

6 DSS Multivariable Cox Models **10**
6.1 Final Baseline Multivariable Cox Model . . . 10
6.2 Final Multivariable Cox Model with CNA Quartiles . . . 11
6.3 Final Multivariable Cox Model with CNA Quartiles and Interactions . . . 11

7 Cox Proportional Hazards Assumption **12**

8 Recursive Partitioning Survival Trees **12**
8.1 Survival Trees with CNA Quartile . . . 12

9 Tishchenko et al Material **13**
9.1 Tishchenko Up-Regulated Genes . . . 13
9.2 Tishchenko Quantiles across CNA Quartiles . . . 13

10 Additional Information **14**
10.1 Contingency Table by PAM50, Grade and CNA Quartile . . . 14

List of Figures

1 Recursive partitioning survival tree considering the six significant clinical variables and CNA Quartiles. . . . 12
2 Stacked barplot showing the proportion and percentage of patients assigned to each Tishchenko quantile within each CNA Quartile in luminal A patients. . . . 13

List of Tables

1 CNA Score ranges corresponding to each CNA Quartile in METABRIC luminal cases. 2
2 List and description of the clinical variables recorded within the METABRIC cohort. 3
3 OS univariate Cox regression on METABRIC clinical characteristics. 4
4 DSS univariate Cox regression on METABRIC clinical characteristics. 5
5 Assessment of association between CNA Quartiles and clinical variables that showed a significant association with OS or DSS in METABRIC luminal cases. 6
6 Clinical characteristics of patients in the METABRIC cohort with luminal breast cancer, classified by CNA Quartiles. 8
7 Clinical characteristics of patients in the METABRIC cohort with luminal A breast cancer, classified by CNA Quartiles. 9
8 Clinical characteristics of patients in the METABRIC cohort with luminal B breast cancer, classified by CNA Quartiles. 9
9 Clinical characteristics of patients in the METABRIC cohort with luminal A histological grade 3 breast cancer, classified by CNA Quartiles. 10
10 Final baseline multivariable Cox model for DSS including clinical variables. 10
11 Final multivariable Cox model for DSS including clinical variables and CNA Quartiles. 11
12 Final multivariable Cox model with selected clinical variables, CNA Quartiles and interactions. 11
13 Assessing the proportional hazards assumption of the final multivariable Cox model 12
14 List of top ten most up-regulated genes in the Tishchenko et al study 13
15 Three-way contingency table containing PAM50, histological grade and CNA Quartile. 14

1 CNA Quartile Cut-Offs

Supplementary Table 1: CNA Score ranges corresponding to each CNA Quartile in METABRIC luminal cases.

	**CNA Score Range**
**CNA Quartile 1**	3–3334
**CNA Quartile 2**	3337–5544
**CNA Quartile 3**	5550–8064
**CNA Quartile 4**	8065–25382

2 Description of Clinical Characteristics

Supplementary Table 2: List and description of the clinical variables recorded within the METABRIC cohort.

Clinical Variable	Description
PAM50 Subtype	The PAM50 classification is based on a 50-gene predictor (PAM50) which measures the expression levels of 50 genes and categorises breast tumours into luminal A, luminal B, HER2-enriched, and basal-like subtypes [1]. The PAM50 classification can provide outcome prediction in both ER-positive and ER-negative tumours [2]. For the purpose of this study only luminal patients within the METABRIC cohort will be analysed.
Integrative Cluster	Dawson et al. [3] used molecular information on the genomic and transcriptomic landscapes of breast cancer to define a genome-driven breast cancer classification. Clustering analysis of joint copy number and gene expression data from the cis-associated genes revealed 10 novel molecular subgroups or Integrative Clusters (IntClusts). Each IntClust has distinct CNAs and gene expression profiles. These clusters were also associated with distinct clinical features and outcomes [3]. Within the METABRIC cohort there are 11 IntClusts due to the splitting of IntClust4 into IntClust4ER- and IntClust4ER+.
Tumour Grade	Tumour grade, commonly referred to as histological grade, is based on the degree of differentiation of the tumour tissue. Histological grade is assessed by the Nottingham Grading System which is based on the evaluation of three morphological features including degree of tubule or gland formation, nuclear pleomorphism, and mitotic count. Tumours can be a well-differentiated tumour (grade 1), a moderately differentiated tumour (grade 2) or a poorly differentiated tumour (grade 3) [4]. Patients within the METABRIC luminal cohort presented with histological grades 1-3.
Tumour Size	The size of the breast tumour measured at its widest point. The tumour size within the METABRIC luminal cohort was measured in millimetres (mm) and ranges from 1-180mm.
Tumour Stage	Tumour stage, commonly referred to as clinical stage, is assessed by the TMN cancer staging system. Clinical stage is defined based on the size of the tumour (T), regional nodal involvement (N), and distant metastasis (M). Stages are generally denoted by Roman numerals from I to IV where higher numbers denote an increase in the extent of disease and corresponding overall prognosis. The term Stage 0 describes carcinoma in situ and is considered to not have metastatic potential [5]. All clinical stages were seen in the METABRIC luminal cohort.
Number of Positive Lymph Nodes	Provides information on the number of positive lymph nodes a patient is found to have. Lymph node involvement is a prognostic factor in breast cancer, where the presence of positive lymph nodes acts as a predictor of increased risk of local and distant recurrence, which directly affects patient outcome [6]. Ranges from 0-45 in the METABRIC luminal cohort.
Nottingham Prognostic Index	The Nottingham Prognostic Index (NPI) is a prognostication tool which aids in the management of breast cancers. It is a prognostic indicator that considers the size of the tumour, the number of lymph nodes involved, and histological grade. It is a continuous score [7] and ranges from 1-6.36 in the METABRIC luminal cohort.
Age at Diagnosis	The age at which a patient is diagnosed with a disease or condition. In this case the age at which the patient was diagnosed with breast cancer. Within the METABRIC luminal cohort patients were between 26 and 92 years of age.
Inferred Menopausal State	Indicates whether women were pre or post-menopausal at diagnosis of breast cancer. The METABRIC luminal cohort contains both pre- and post-menopausal women.
ER Status	Estrogen receptors (ERs) are hormone receptors found on breast cells which receive signals from estrogen which may promote its growth. Breast tumours are categorised as ER-positive (ER+) if they have receptors for the hormone estrogen and ER-negative (ER-) if they do not. ER receptor status is a strong predictive biomarker. If a patient's tumor expresses ERs they may positively benefit from endocrine therapy such as tamoxifen [8]. Varying methods can be used to determine ER status, including ER immunohistochemistry and mRNA expression [9]. In this case ER status was determined by mRNA expression as positive or negative. The METABRIC luminal cohort contains both ER+ and ER- cases.
ER Immunohistochemistry	The presence of ERs as detected by immunohistochemistry (IHC). ER status refers to whether a patient is clinically categorised as ER-positive (ER+), if they have receptors for the hormone estrogen, or ER-negative (ER-) if they do not. ER IHC is the most commonly used method of testing for ER status. According to the American Society for Clinical Oncology/College of American Pathologists criteria for ER positivity by IHC, a patient is categorised as ER+ if greater than 1% of the cells stain positive for ER. Generally ER determination using microarray gene expression and IHC have high concordance rates [10].
PR Status	Progesterone receptors (PRs) are hormone receptors found on breast cells which receive signals from progesterone which could promote its growth. Breast tumours are categorised as PR-positive (PR+) if they have receptors for the hormone progesterone and PR-negative (PR-) if they do not. PR receptor status is a strong predictive biomarker. If a patient's tumor expresses PRs they may positively benefit from endocrine therapy such as tamoxifen [8]. The METABRIC luminal cohort contains both PR+ and PR- cases.
HER2 Status	Human epidermal growth factor 2 (HER2) is on chromosome 17q12. This gene is amplified in approximately 15-25% of breast cancers and can activate several signaling pathways leading to increased cell proliferation. HER2 status informs whether the HER2 gene is amplified in breast cancer and can act as an important prognostic indicator [8, 11]. Varying methods can be used to determine HER2 status, including IHC, which tests for protein expression, or fluorescence in situ hybridization (FISH), which tests for gene amplification. In this case HER2 status was determined by mRNA expression [12, 9]. METABRIC luminal patients were categorised as being HER2- (no amplification) or HER2+ (HER2 amplified).
HER2 SNP6	HER2 status determined through the use of high-density single nucleotide polymorphism (SNP) arrays, in this case the Affymetrix SNP6 array. These arrays detect copy number variations (CNVs) i.e. amplifications or deletions. Amplifications may increase HER2 gene expression resulting in the tumour being categorised as HER2 positive [11]. HER2 SNP6 is categorised as gain, loss, neutral or undefined in the METABRIC luminal cohort.
Three Gene Classification	Samples were classified into the four Gene Expression progNostic Index Using Subtypes (GENIUS) subgroups which included HER2+, ER-/HER2-, ER+/HER2- high proliferation and ER+/HER2- low proliferation. This was carried out by Curtis et al. [9] using the genefu package in Bioconductor. The METABRIC luminal cohort contains all subgroups.
Cellularity	Tumour cellularity is the relative proportion of tumour and normal cells in a sample. Tumour cellularity can affect the sensitivity of processes such as mutation detection and copy number analysis [4]. In the METABRIC study tumour cellularity was scored visually in a semi-quantitative fashion. Cellularity values were binned into low cellularity (<40% tumour DNA), moderate cellularity (40% - 70% tumour DNA), and high cellularity (>70% tumour DNA) [9].
Laterality	This is the location of primary tumour. Primary tumour laterality can be classified as left side, right side, or bilateral [13]. In the METABRIC luminal cohort patients presented with either left or right side laterality.
Histological Subtype	Breast cancer can be classified and sub-classified based on histopathologic characteristics, including cell morphology, architecture, and growth patterns, into 21 distinct subtypes [14]. The METABRIC luminal cohort contains the following histological subtypes: Ductal/No Special Type, Lobular, Medullary, Mixed, Mucinous, Other and Tubular/Cribriform.
Cancer Type Detailed	Indicates whether the breast cancers begin in the cells that line the ducts (ductal cancers), in the cells that line the lobules (lobular cancers) or in other tissues [15]. Within the METABRIC luminal cohort patients presented with Breast, Breast Invasive Ductal Carcinoma, Breast Invasive Lobular Carcinoma, Breast Invasive Mixed Mucinous Carcinoma and Breast Mixed Ductal and Lobular Carcinoma.
Chemotherapy	Cancer chemotherapy refers to the administration of cytotoxic chemicals with the aim to kill or at least shrink the tumour. Cytotoxic drugs are mostly given by the intravenous route [16]. This variable indicates whether a patient received chemotherapy as part of their treatment regime (Yes or No).
Radiotherapy	Radiation therapy (Radiotherapy) is the application of high energy X-rays to shrink a tumour. Radiotherapy aims to deprive tumour cells of their multiplication potential. High-energy radiation does this by inducing genetic damage, leading to cell death. Radiotherapy is an important part of cancer treatment with about 50% of all cancer patients receiving radiotherapy [17]. This clinical variable indicates whether a patient received radiotherapy as part of their treatment regime (Yes or No).
Hormone Therapy	Endocrine therapy, also known as hormone therapy, lowers estrogen levels and inhibits the growth of the cancer. Hormone therapy is a systemic treatment for hormone receptor-positive (ER+ and PR+) breast cancer in the adjuvant, metastatic, and sometimes neoadjuvant setting [18]. This variable records whether a patient received hormone therapy as part of their treatment regime (Yes or No).
Breast Surgery	Whether the patient had breast-conserving surgery (BCS) (also known as lumpectomy) or a mastectomy. A mastectomy involves removal of the entire breast, while in BCS, the cancerous tumour is removed along with a margin of non-cancerous breast tissue [19]. Patients within the METABRIC luminal cohort were classified as having had BCS or a mastectomy.

3 Univariate Cox Models

3.1 Overall Survival

Supplementary Table 3: Univariate Cox regression on METABRIC clinical characteristics with p-values adjusted for multiple testing using the Benjamini-Hochberg procedure. Rows where univariate Cox regression had a significant global adjusted p-value for OS are highlighted. Note that both the adjusted Wald test and adjusted likelihood ratio test p-values were used to determine significance. This was due to some factor level sample sizes being sufficiently small.

Clinical Variable	Hazard Ratio	Hazard Ratio (CILL)	Hazard Ratio (CIUL)	P-value (Wald Test/LRT)	Adjusted P-value
PAM50:				3.93e-09/5.72e-09	1.13e-08/1.65e-08
Luminal A (Ref)	-	-	-	-	-
Luminal B	1.569e+00	1.351e+00	1.823e+00	3.925e-09	-
Integrative Cluster:				9.45e-07/3.1e-06	2.41e-06/7.14e-06
1 (Ref)	-	-	-	-	-
2	1.188e+00	7.984e-01	1.767e+00	3.955e-01	-
3	7.249e-01	5.308e-01	9.899e-01	4.302e-02	-
4ER-	5.157e-06	0.000e+00	Inf	9.881e-01	-
4ER+	6.445e-01	4.454e-01	9.327e-01	1.982e-02	-
5	1.821e+00	1.205e+00	2.752e+00	4.409e-03	-
6	1.014e+00	6.828e-01	1.505e+00	9.467e-01	-
7	8.100e-01	5.845e-01	1.122e+00	2.055e-01	-
8	8.287e-01	6.147e-01	1.117e+00	2.176e-01	-
9	1.266e+00	8.848e-01	1.812e+00	1.970e-01	-
10	4.305e-01	1.841e-01	1.006e+00	5.173e-02	-
Histological Grade:				1.28e-04/1.15e-04	2.27e-04/2.03e-04
1 (Ref)	-	-	-	-	-
2	1.264e+00	9.647e-01	1.657e+00	8.925e-02	-
3	1.655e+00	1.258e+00	2.177e+00	3.191e-04	-
Tumour Size	1.019e+00	1.015e+00	1.023e+00	1.91e-24/2.79e-16	2.2e-23/2.14e-15
Clinical Stage:				2.61e-14/8.53e-14	1.2e-13/3.92e-13
0	8.230e-06	0.000e+00	Inf	9.888e-01	-
1 (Ref)	-	-	-	-	-
2	1.843e+00	1.514e+00	2.242e+00	1.034e-09	-
3	3.126e+00	2.222e+00	4.398e+00	5.864e-11	-
4	6.218e+00	2.902e+00	1.332e+01	2.597e-06	-
Positive Lymph Nodes	1.059e+00	1.046e+00	1.072e+00	1.2e-20/3.44e-13	9.24e-20/1.32e-12
NPI	1.328e+00	1.239e+00	1.424e+00	1.08e-15/1.5e-15	6.2e-15/8.6e-15
Age at Diagnosis	1.055e+00	1.047e+00	1.063e+00	7.77e-47/5.02e-51	1.79e-45/1.15e-49
Inferred Menopausal State:				3.64e-13/4.42e-17	1.4e-12/5.08e-16
Pre (Ref)	-	-	-	-	-
Post	2.809e+00	2.126e+00	3.711e+00	3.640e-13	-
ER Status:				8.16e-01/8.21e-01	8.16e-01/8.21e-01
Negative (Ref)	-	-	-	-	-
Positive	8.481e-01	2.115e-01	3.400e+00	8.162e-01	-
ER Immunohistochemistry				2.63e-02/4.04e-02	3.56e-02/4.89e-02
Negative (Ref)	-	-	-	-	-
Positive	5.880e-01	3.681e-01	9.393e-01	2.629e-02	-
PR Status:				1.24e-05/1.97e-05	2.37e-05/3.78e-05
Negative (Ref)	-	-	-	-	-
Positive	6.989e-01	5.952e-01	8.207e-01	1.238e-05	-
HER2 Status:				7.1e-04/1.67e-03	1.17e-03/2.75e-03
Negative (Ref)	-	-	-	-	-
Positive	1.697e+00	1.249e+00	2.304e+00	7.099e-04	-
HER2 SNP6:				2.16e-02/2.72e-02	3.11e-02/3.68e-02
Loss(Ref)	-	-	-	-	-
Neutral	1.088e+00	7.693e-01	1.540e+00	6.327e-01	-
Gain	1.463e+00	1.000e+00	2.140e+00	4.976e-02	-
Undefined	7.385e-01	1.773e-01	3.075e+00	6.770e-01	-
Three Gene Classification:				1.5e-06/4.43e-06	3.14e-06/9.26e-06
ER-/HER2- (Ref)	-	-	-	-	-
ER+/HER2- Low Prolif	7.609e-02	1.058e-02	5.471e-01	1.049e-02	-
ER+/HER2- High Prolif	1.118e-01	1.556e-02	8.025e-01	2.935e-02	-
HER2+	1.303e-01	1.764e-02	9.621e-01	4.573e-02	-
Cellularity:				4.56e-01/4.54e-01	4.77e-01/4.74e-01
Low (Ref)	-	-	-	-	-
Moderate	1.060e+00	7.630e-01	1.472e+00	7.284e-01	-
High	1.154e+00	8.356e-01	1.593e+00	3.849e-01	-
Laterality:				4.36e-01/4.36e-01	4.77e-01/04.74e-01
Left (Ref)	-	-	-	-	-
Right	9.409e-01	8.071e-01	1.097e+00	4.362e-01	-
Histological Subtype:				4.06e-02/2.39e-03	5.19e-02/3.66e-03
Ductal/NST (Ref)	-	-	-	-	-
Lobular	1.029e+00	7.694e-01	1.376e+00	8.480e-01	-
Medullary	1.081e+00	1.519e-01	7.693e+00	9.380e-01	-
Mixed	1.057e+00	8.570e-01	1.305e+00	6.025e-01	-
Mucinous	6.580e-01	3.269e-01	1.324e+00	2.408e-01	-
Other	2.105e-01	5.250e-02	8.444e-01	2.791e-02	-
Tubular/Cribiform	3.173e-01	1.314e-01	7.660e-01	1.068e-02	-
Cancer Type Detailed				1.54e-01/3.2e-02	1.78e-01/4.08e-02
Invasive Ductal Carcinoma (Ref)	-	-	-	-	-
Breast	2.153e-01	5.368e-02	8.634e-01	3.021e-02	-
Invasive Lobular Carcinoma	1.050e+00	7.855e-01	1.404e+00	7.409e-01	-
Invasive Mixed Mucinous Carcinoma	6.732e-01	3.345e-01	1.355e+00	2.675e-01	-
Mixed Ductal and Lobular Carcinoma	1.079e+00	8.751e-01	1.332e+00	4.752e-01	-
Chemotherapy:				4.63e-02/5.52e-04	5.6e-02/6.35e-02
No (Ref)	-	-	-	-	-
Yes	1.333e+00	1.005e+00	1.769e+00	4.628e-02	-

Hormone Therapy:				1.4e-06/6.64e-07	3.14e-06/1.7e-06
No (Ref)	-	-	-	-	-
Yes	1.528e+00	1.286e+00	1.816e+00	1.403e-06	-
Radiotherapy:				2.07e-02/2.1e-02	3.11e-02/3.02e-02
No (Ref)	-	-	-	-	-
Yes	8.386e-01	7.223e-01	9.735e-01	2.073e-02	-
Breast Surgery:				2.66e-09/1.2e-09	8.74e-09/3.94e-09
Breast Conserving (Ref)	-	-	-	-	-
Mastectomy	1.616e+00	1.380e+00	1.893e+00	2.661e-09	-
CILL: Confidence Interval Lower Limit; CIUL: Confidence Interval Upper Limit Interval; LRT: Likelihood ratio test; Ref; Reference level; NPI: Nottingham Prognostic Index; ER: Estrogen receptor; PR: Progesterone receptor; HER2: Human epidermal growth factor receptor 2; NST: No special type.					

3.2 Disease-Specific Survival

Supplementary Table 4: Univariate Cox regression on METABRIC clinical characteristics with p-values adjusted for multiple testing using the Benjamini-Hochberg procedure. Rows where univariate Cox regression had a significant global adjusted p-value for DSS are highlighted. Note that both the adjusted Wald test and adjusted likelihood ratio test p-values were used to determine significance. This was due to some factor level sample sizes being sufficiently small.

Clinical Variable	Hazard Ratio	Hazard Ratio (CILL)	Hazard Ratio (CIUL)	P-value (Wald Test/LRT)	Adjusted P-value
PAM50:				2.93e-13/2.83e-13	1.35e-12/1.63e-12
Luminal A (Ref)	-	-	-	-	-
Luminal B	2.218e+00	1.791e+00	2.747e+00	2.928e-13	-
Integrative Cluster:				4.2e-12/2.22e-11	1.61e-11/8.5e-11
1 (Ref)	-	-	-	-	-
2	1.308e+00	8.043e-01	2.127e+00	2.793e-01	-
3	3.839e-01	2.461e-01	5.989e-01	2.454e-05	-
4ER-	3.580e-06	0.000e+00	Inf	9.908e-01	-
4ER+	4.533e-01	2.709e-01	7.586e-01	2.601e-03	-
5	1.892e+00	1.145e+00	3.126e+00	1.278e-02	-
6	1.030e+00	6.265e-01	1.693e+00	9.074e-01	-
7	5.455e-01	3.478e-01	8.556e-01	8.312e-03	-
8	6.074e-01	4.101e-01	8.995e-01	1.283e-02	-
9	1.142e+00	7.237e-01	1.802e+00	5.685e-01	-
10	5.055e-01	1.797e-01	1.422e+00	1.961e-01	-
Histological Grade:				2.01e-08/2.82e-09	5.77e-08/8.11e-09
1 (Ref)	-	-	-	-	-
2	2.033e+00	1.246e+00	3.316e+00	4.477e-03	-
3	3.353e+00	2.060e+00	5.457e+00	1.128e-06	-
Tumour Size	1.022e+00	1.017e+00	1.026e+00	6.8e-23/6.76e-14	5.21e-22/7.78e-13
Clinical Stage:				1.1e-13/6.37e-12	6.32e-13/2.93e-11
0	5.895e-06	0.000e+00	Inf	9.923e-01	-
1 (Ref)	-	-	-	-	-
2	1.964e+00	1.479e+00	2.609e+00	3.174e-06	-
3	4.194e+00	2.723e+00	6.459e+00	7.611e-11	-
4	1.073e+01	4.904e+00	2.346e+01	2.817e-09	-
Positive Lymph Nodes	1.072e+00	1.057e+00	1.087e+00	5.55e-23/2.01e-13	5.21e-22/1.54e-12
NPI	1.656e+00	1.502e+00	1.826e+00	3.66e-24/4.24e-24	8.43e-23/9.76e-23
Age at Diagnosis	1.022e+00	1.013e+00	1.032e+00	6.21e-06/4.61e-06	1.3e-05/1.18e-05
Inferred Menopausal State:				1.55e-02/1.12e-02	2.1e-02/1.51e-02
Pre (Ref)	-	-	-	-	-
Post	1.480e+00	1.077e+00	2.033e+00	1.55e-02	-
ER Status:				9.74e-01/9.74e-01	9.74e-01/9.74e-01
Negative (Ref)	-	-	-	-	-
Positive	9.679e-01	1.359e-01	6.895e+00	9.741e-01	-
ER Immunohistochemistry:				1.32e-03/4.88e-03	2.02e-03/7.01e-03
Negative (Ref)	-	-	-	-	-
Positive	4.028e-01	2.313e-01	7.016e-01	1.318e-03	-
PR Status:				5.03e-06/9.24e-06	1.16e-05/1.93e-05
Negative (Ref)	-	-	-	-	-
Positive	5.967e-01	4.781e-01	7.449e-01	5.033e-06	-
HER2 Status:				6.97e-06/5.85e-05	1.34e-05/1.03e-04
Negative (Ref)	-	-	-	-	-
Positive	2.337e+00	1.614e+00	3.384e+00	6.974e-06	-
HER2 SNP6:				1.33e-02/1.93e-02	1.91e-02/2.47e-02
Loss (Ref)	-	-	-	-	-
Neutral	8.494e-01	5.385e-01	1.340e+00	4.829e-01	-
Gain	1.322e+00	8.034e-01	2.174e+00	2.722e-01	-
Undefined	6.436e-01	8.632e-02	4.798e+00	6.672e-01	-
Three Gene Classification:				9.48e-11/1.94e-10	3.11e-10/6.37e-10
ER-/HER2- (Ref)	-	-	-	-	-
ER+/HER2- Low Prolif	4.079e-02	5.621e-03	2.960e-01	1.558e-03	-
ER+/HER2- High Prolif	8.494e-02	1.177e-02	6.132e-01	1.449e-02	-
HER2+	1.236e-01	1.648e-02	9.268e-01	4.196e-02	-
Cellularity:				1.33e-01/1.09-01	1.7e-01/1.32e-01
Low (Ref)	-	-	-	-	-
Moderate	1.460e+00	8.414e-01	2.533e+00	1.784e-01	-
High	1.659e+00	9.639e-01	2.854e+00	6.765e-02	-
Laterality:				3.98e-01/3.97e-01	4.81e-01/4.35e-01
Left (Ref)	-	-	-	-	-
Right	9.096e-01	7.302e-01	1.133e+00	3.977e-01	-
Histological Subtype:				6.9e-01/3.32e-03	7.56e-01/5.09e-03
Ductal/NST (Ref)	-	-	-	-	-
Lobular	9.673e-01	6.367e-01	1.470e+00	8.763e-01	-
Medullary	1.937e+00	2.717e-01	1.381e+01	5.094e-01	-
Mixed	9.310e-01	6.823e-01	1.270e+00	6.518e-01	-
Mucinous	5.357e-01	1.715e-01	1.673e+00	2.828e-01	-

Other	2.290e-01	3.212e-02	1.632e+00	1.413e-01	-
Tubular/Cribiform	1.045e-07	0.000e+00	Inf	9.888e-01	-
Cancer Type Detailed:				5.31e-01/3.07e-01	6.11e-01/3.53e-01
Invasive Ductal Carcinoma (Ref)	-	-	-	-	-
Breast	2.354e-01	3.302e-02	1.678e+00	1.489e-01	-
Invasive Lobular Carcinoma	9.924e-01	6.533e-01	1.508e+00	9.715e-01	-
Invasive Mixed Mucinous Carcinoma	5.509e-01	1.764e-01	1.721e+00	3.048e-01	-
Mixed Ductal and Lobular Carcinoma	9.553e-01	7.003e-01	1.303e+00	7.731e-01	-
Chemotherapy:				8.38e-07/7.13e-06	2.14e-06/1.64e-05
No(Ref)	-	-	-	-	-
Yes	2.200e+00	1.608e+00	3.011e+00	8.385e-07	-
Hormone Therapy:				6.275e-04/3.99e-04	1.03e-03/6.55e-04
No(Ref)	-	-	-	-	-
Yes	1.553e+00	1.207e+00	1.999e+00	6.275e-04	-
Radiotherapy:				9.44e-01/9.44e-01	9.74e-01/9.74e-01
No(Ref)	-	-	-	-	-
Yes	9.924e-01	8.007e-01	1.230e+00	9.441e-01	-
Breast Surgery:				6.06e-05/4.17e-05	1.07e-04/8e-05
Breast Conserving (Ref)	-	-	-	-	-
Mastectomy	1.589e+00	1.267e+00	1.992e+00	6.065e-05	-
CILL: Confidence Interval Lower Limit; CIUL: Confidence Interval Upper Limit Interval; LRT: Likelihood ratio test; Ref; Reference level; NPI: Nottingham Prognostic Index; ER: Estrogen receptor; PR: Progesterone receptor; HER2: Human epidermal growth factor receptor 2; NST: No special type.					

4 Association Analysis

Supplementary Table 5: Assessment of association between CNA Quartiles and clinical variables that showed a significant association with OS or DSS in METABRIC luminal cases. The Benjamini-Hochberg procedure was used to correct for multiple testing. Adjusted p-values based on Chi-Squared and Kruskal-Wallis tests are shown and significant p-values are highlighted.

Clinical Variable	Chi-Squared Test	Fisher's Exact Test	Kruskal-Wallis Test	Adjusted P-value
PAM50	7.397e-30	4.998e-04'		3.698e-29
Integrative Cluster	4.252e-60*	4.998e-04'		8.504e-59
Histological Grade	4.083e-32	4.998e-04'		2.722e-31
Tumour Size			1.701e-10	4.861e-10
Clinical Stage	6.971e-04*	4.998e-04'		9.959e-04
Positive Lymph Nodes			4.844e-04	7.452e-04
NPI			2.887e-24	1.155e-23
Age at Diagnosis			1.376e-02	1.619e-02
Inferred Menopausal State	6.567e-02	6.265e-02		7.296e-02
ER Immunohistochemistry	5.055e-01	4.841e-01		5.321e-01
PR Status	2.752e-10	5.326e-11		6.879e-10
HER2 Status	5.036e-06	3.192e-06		1.007e-05
HER2 SNP6	1.531e-10*	4.998e-04'		4.861e-10
Three Gene Classification	4.535e-42*	4.998e-04'		4.535e-41
Histological Subtype	8.549e-08*	4.998e-04'		1.900e-07
Cancer Type Deatiled	3.310e-04*	9.995e-04'		5.516e-04
Chemotherapy	3.964e-03	2.314e-03		5.285e-03
Hormone Therapy	4.264e-05	4.789e-05		7.753e-05
Radiotherapy	1.039e-02	1.091e-02		1.299e-02
Breast Surgery	5.600e-01	5.669e-01		5.600e-01
* Chi-Squared approximation may be incorrect<br>' Simulated P-value for Fisher's Exact Test<br>NPI: Nottingham Prognostic Index; ER: Estrogen receptor; HER2: Human epidermal growth factor receptor 2;<br>PR: Progesterone receptor				

5 Clinical Variable Selection and Summaries

5.1 Clinical Variable Selection

We carried out a univariate Cox regression for each of the clinical variables described in Supplementary Table 2 and subsequently determined if any of the OS or DSS associated clinical variables were also associated with the CNA Quartiles. Clinical variables associated with OS and DSS which were also associated with our CNA Quartiles included PAM50 Subtype, Integrative Cluster, Histological Grade, Tumour Size, Clinical Stage, Positive Lymph Nodes, NPI, Age at Diagnosis, PR Status, HER2 Status, HER2 SNP6, Three Gene Classification, Histological Subtype and Hormone Therapy. Clinical variables associated solely with OS and CNA Quartiles include Cancer Type Detailed and Radiotherapy, while clinical variables associated solely with DSS and CNA Quartiles include Chemotherapy.

Before constructing multivariable Cox models for OS and DSS we carried out clinical variable selection to remove high correlations among the predictor variables. This was because high correlations among predictor variables may lead to unreliable and unstable estimates of regression coefficients. The clinical variables considered for selection are as follows:

1.) Integrative cluster: Within the METABRIC cohort two breast cancer classifications are recorded - PAM50 Subtype and Integrative Cluster. Clustering analysis of joint copy number and gene expression data from the cis-associated genes revealed 10 novel molecular subgroups or Integrative Clusters (IntClusts) [3]. Each of these IntClusts have distinct CNAs and gene expression profiles. Although PAM50 and IntClusts are different methods of breast cancer classification, associations between the two were observed, with luminal A and luminal B cases being more likely to fall into certain IntClusts. For example, IntClusts 1 and 6 predominantly contain luminal B cases, while IntClusts 3 and 7 are primarily composed of luminal A cases [3]. As a result of this association and the fact PAM50 Subtype is a clinically utilised classification, the decision was made to exclude Integrative cluster from the analysis and focus on PAM50 Subtype.

2.) Clinical stage: Clinical stage is determined using the TNM system which classifies cancers by the size and extent of the primary tumor (T), involvement of regional lymph nodes (N), and the presence or absence of distant metastases (M). For some cancer types, in addition to T, N, and M categories, prognostic factors are required to assign a stage group. Examples include histological grade, age at diagnosis, histological type, mitotic rate, serum tumor markers, hormone receptors [5]. As such, within the METABRIC cohort clinical stage is directly associated with the clinical variables tumour size and number of lymph nodes positive and possibly other variables like histological grade. Due to this clinical stage was excluded from the analysis.

3.) NPI: NPI is a prognostic indicator that takes into account the size of the tumour, the number of lymph nodes involved, and the histological grade [7]. The decision was made to utilise the three components of NPI rather than NPI itself in order to investigate the relationship between the CNA Quartiles and a number of widely used clinical variables such as histological grade.

4.) HER SNP6: The HER2 Status and HER2 SNP6 clinical variables recorded within the METABRIC cohort are seen to be correlated with each other. HER2 Status was determined by mRNA expression where each patient was categorised as HER2- or HER2+. However, HER2 Status can also be determined through the use of high-density single nucleotide polymorphism (SNP) arrays. Within the METABRIC cohort they utilised the Affymetrix SNP6 array [9] to produce the variable HER2 SNP6. These arrays detect CNVs which can determine whether there is a copy number change associated with HER2. HER2 SNP6 can be categorised as gain, loss, neutral or undefined. Tumours possessing a HER2 gain may have higher expression of HER2 and as such are more likely to be categorised as HER2+. As these two clinical variables aim to capture similar information, and as such are correlated, the decision was made to exclude HER2 SNP6 from

our analysis. Reasons for this include that HER Status provides binary information on HER2 positivity and that the other two breast cancer biomarkers measured in the METABRIC cohort i.e. ER Status and PR Status were also determined by mRNA expression [9]. Therefore, in the interest of clarity and consistency HER2 Status was used.

5.) Three Gene Classification: Three Gene Classification was determined by classifying samples into the four Gene Expression progNostic Index Using Subtypes (GENIUS) subgroups using the genefu package in Bioconductor. These subgroups included HER2+, ER-/HER2-, ER+/HER2- high proliferation and ER+/HER2- low proliferation [9]. As the Three Gene Classification utilises HER Status and ER Status it was seen to be highly correlated with the HER2 and ER Status clinical variables. Three gene classification is also seen to be associated with PAM50 Subtype as luminal B tumours are more likely to have higher expression of proliferation/cell cycle-related genes or proteins (e.g., MKI67 and AURKA) [20] and as such are more likely to be cateogrised as ER+/HER2- high proliferation. To eliminate these correlations Three Gene Classification was excluded from the downstream analysis.

6.) Treatment: Other clinical variables that were not considered in the analysis include the treatment variables i.e. Chemotherapy, Radiotherapy, Hormone Therapy and Breast Surgery.

5.2 Selected Clinical Variable Summaries

5.2.1 All Luminal Cases

Supplementary Table 6: Clinical characteristics of patients in the METABRIC cohort with luminal breast cancer, classified by CNA Quartiles.

Clinical Variable	All Cases (N = 1,175)	Quartile 1 (N = 294)	Quartile 2 (N = 294)	Quartile 3 (N = 293)	Quartile 4 (N = 294)
**Subtype**					
Luminal A	700 (59.57%)	228 (77.55%)	207 (70.41%)	167 (57.00%)	98 (33.33%)
Luminal B	475 (40.43%)	66 (22.45%)	87 (29.59%)	126 (43.00%)	196 (66.67%)
**Histological Grade**					
1	136 (11.57%)	61 (20.75%)	43 (14.63%)	22 (7.51%)	10 (3.40%)
2	564 (48.00%)	163 (55.44%)	168 (57.14%)	138 (47.10%)	95 (32.31%)
3	425 (36.17%)	52 (17.69%)	70 (23.81%)	125 (42.66%)	178 (60.54%)
**Tumour Size**					
mean	25.4	22.49	25.72	25.53	27.86
median (IQR)	22.00 (17.00, 30.00)	20.00 (15.00, 25.00)	23.00 (16.00, 30.00)	22.00 (18.00, 30.00)	25.00 (20.00, 33.00)
**Lymph Nodes Positive**					
mean	1.8	1.06	1.89	1.97	2.24
median (IQR)	0.00 (0.00, 2.00)	0.00 (0.00, 1.00)	0 (0.00, 2.00)	0 (0.00, 2.00)	1.00 (0.00, 2.00)
$\geq$ 1 Lymph node positive	524 (45.96%)	102 (37.23%)	138 (48.76%)	134 (46.37%)	150 (51.02%)
**Age at Diagnosis**					
mean	63.86	64.19	64.51	61.72	65.01
median (IQR)	64.76 (55.56, 72.78)	65.33 (55.41, 73.47)	66.22 (56.12, 74.18)	62.88 (52.79, 71.17)	65.45 (58.45, 72.60)
**HER2 Status**					
Negative	1,109 (94.38%)	288 (97.96%)	287 (97.62%)	270 (92.15%)	264 (89.80%)
Positive	66 (5.62%)	6 (2.04%)	7 (2.38%)	23 (7.85%)	30 (10.20%)
**PR Status**					
Negative	337 (28.68%)	42 (14.29%)	81 (27.55%)	109 (37.20%)	105 (35.71%)
Positive	838 (71.32%)	252 (85.71%)	213 (72.45%)	184 (62.80%)	189 (64.29%)
**Histological Subtype**					
Ductal/NST	864 (73.53%)	190 (64.63%)	203 (69.05%)	221 (75.43%)	250 (85.03%)
Lobular	86 (7.32%)	23 (7.82%)	26 (8.84%)	26 (8.87%)	11 (3.74%)
Medullary	2 (0.17%)	1 (0.34%)	0 (0.00%)	0 (0.00%)	1 (0.34%)
Mixed	160 (13.62%)	45 (15.31%)	51 (17.35%)	37 (12.63%)	27 (9.18%)
Mucinous	20 (1.70%)	11 (3.74%)	5 (1.70%)	2 (0.68%)	2 (0.68%)
Tubular/Cribriform	18 (1.53%)	14 (4.76%)	2 (0.68%)	2 (0.68%)	0 (0.00%)
Other	10 (0.85%)	4 (1.36%)	1 (0.34%)	4 (1.37%)	1 (0.34%)

IQR: Interquartile range; HER2: Human epidermal growth factor receptor 2; PR: Progesterone receptor; NST: No special type.

5.2.2 Luminal A Cases

Supplementary Table 7: Clinical characteristics of patients in the METABRIC cohort with luminal A breast cancer, classified by CNA Quartiles.

Clinical Variable	Luminal A (N = 700)	Quartile 1 (N = 228)	Quartile 2 (N = 207)	Quartile 3 (N = 167)	Quartile 4 (N = 98)
**Subtype**					
Luminal A	700 (100.00%)	228 (100.00%)	207 (100.00%)	167 (100.00%)	98 (100.00%)
Luminal B	0 (0.00%)	0 (0.00%)	0 (0.00%)	0 (0.00%)	0 (0.00%)
**Histological Grade**					
1	118 (16.86%)	54 (23.68%)	38 (18.36%)	19 (11.38%)	7 (7.14%)
2	378 (54.00%)	129 (56.58%)	119 (57.49%)	85 (50.90%)	45 (45.92%)
3	172 (24.57%)	30 (13.16%)	41 (19.81%)	59 (35.33%)	42 (42.86%)
**Tumour Size**					
mean	23.95	21.51	24.41	24.65	27.46
median (IQR)	20.00 (16.00, 29.00)	20.00 (15.00, 25.00)	21.60 (16.00, 28.50)	21.00 (16.10, 30.00)	25.00 (20.00, 30.00)
**Lymph Nodes Positive**					
mean	1.5	1.00	1.54	1.75	2.08
median (IQR)	0 (0.00, 2.00)	0 (0.00, 1.00)	0.00 (0.00, 2.00)	0.00 (0.00, 2.00)	0.00 (0.00, 3.00)
$\geq$ 1 Lymph node positive	287 (42.27%)	82 (38.14%)	92 (46.00%)	67 (40.36%)	46 (46.94%)
**Age at Diagnosis**					
mean	62.86	63.39	63.67	59.84	65.04
median (IQR)	63.37 (53.17, 72.25)	64.12 (53.98, 72.25)	64.40 (54.48, 74.08)	58.78 (50.92, 69.66)	65.28 (58.26, 72.30)
**HER2 Status**					
Negative	679 (97.00%)	224 (98.25%)	203 (98.07%)	159 (95.21%)	93 (94.90%)
Positive	21 (3.00%)	4 (1.75%)	4 (1.93%)	8 (4.79%)	5 (5.10%)
**PR Status**					
Negative	161 (23.00%)	30 (13.16%)	48 (23.19%)	60 (35.93%)	23 (23.47%)
Positive	539 (77.00%)	198 (86.84%)	159 (76.81%)	107 (64.07%)	75 (76.53%)
**Histological Subtype**					
Ductal/NST	487 (69.57%)	147 (64.47%)	140 (67.63%)	121 (72.46%)	79 (80.61%)
Lobular	64 (9.14%)	19 (8.33%)	23 (11.11%)	19 (11.38%)	3 (3.06%)
Medullary	0 (0.00%)	0 (0.00%)	0 (0.00%)	0 (0.00%)	0 (0.00%)
Mixed	109 (15.57%)	32 (14.04%)	39 (18.84%)	23 (13.77%)	15 (15.31%)
Mucinous	8 (1.14%)	7 (3.07%)	1 (0.48%)	0 (0.00%)	0 (0.00%)
Tubular/Cribriform	18 (2.57%)	14 (6.14%)	2 (0.97%)	2 (1.20%)	0 (0.00%)
Other	5 (0.71%)	4 (1.75%)	0 (0.00%)	1 (0.60%)	0 (0.00%)

IQR: Interquartile range; HER2: Human epidermal growth factor receptor 2; PR: Progesterone receptor; NST: No special type.

5.2.3 Luminal B Cases

Supplementary Table 8: Clinical characteristics of patients in the METABRIC cohort with luminal B breast cancer, classified by CNA Quartiles.

Clinical Variable	Luminal B (N = 475)	Quartile 1 (N = 66)	Quartile 2 (N = 87)	Quartile 3 (N = 126)	Quartile 4 (N = 196)
**Subtype**					
Luminal A	0 (0.00%)	0 (0.00%)	0 (0.00%)	0 (0.00%)	0 (0.00%)
Luminal B	475 (100.00%)	66 (100.00%)	87 (100.00%)	126 (100.00%)	196 (100.00%)
**Histological Grade**					
1	18 (3.79%)	7 (10.61%)	5 (5.75%)	3 (2.38%)	3 (1.53%)
2	186 (39.16%)	34 (51.52%)	49 (56.32%)	53 (42.06%)	50 (25.51%)
3	253 (53.26%)	22 (33.33%)	29 (33.33%)	66 (52.38%)	136 (69.39%)
**Tumour Size**					
mean	27.55	25.88	28.87	26.71	28.06
median (IQR)	25.00 (20.00, 31.00)	21.00 (18.00, 30.00)	25.00 (19.25, 32.00)	25.00 (18.82, 30.00)	25.00 (20.00, 33.75)
**Lymph Nodes Positive**					
mean	2.24	1.25	2.72	2.26	2.32
median (IQR)	1 (0.00, 2.00)	0 (0.00, 1.00)	1 (0.00, 3.00)	1 (0.00, 2.00)	1.00 (0.00, 2.00)
$\geq$ 1 Lymph node positive	237 (51.41%)	20 (33.90%)	46 (55.42%)	67 (54.47%)	104 (53.06%)
**Age at Diagnosis**					
mean	65.33	66.94	66.50	64.21	65.00
median (IQR)	66.52 (58.89, 73.96)	67.87 (59.20, 75.47)	68.42 (60.21, 74.48)	65.90 (57.22, 72.09)	65.53 (58.66, 73.44)
**HER2 Status**					
Negative	430 (90.53%)	64 (96.97%)	84 (96.55%)	111 (88.10%)	171 (87.24%)
Positive	45 (9.47%)	2 (3.03%)	3 (3.45%)	15 (11.90%)	25 (12.76%)
**PR Status**					
Negative	176 (37.05%)	12 (18.18%)	33 (37.93%)	49 (38.89%)	82 (41.84%)
Positive	299 (62.95%)	54 (81.82%)	54 (62.07%)	77 (61.11%)	114 (58.16%)
**Histological Subtype**					
Ductal/NST	377 (79.37%)	43 (65.15%)	63 (72.41%)	100 (79.37%)	171 (87.24%)
Lobular	22 (4.63%)	4 (6.06%)	3 (3.45%)	7 (5.56%)	8 (4.08%)
Medullary	2 (0.42%)	1 (1.52%)	0 (0.00%)	0 (0.00%)	1 (0.51%)
Mixed	51 (10.74%)	13 (19.70%)	12 (13.79%)	14 (11.11%)	12 (6.12%)
Mucinous	12 (2.53%)	4 (6.06%)	4 (4.60%)	2 (1.59%)	2 (1.02%)
Tubular/Cribriform	0 (0.00%)	0 (0.00%)	0 (0.00%)	0 (0.00%)	0 (0.00%)
Other	5 (1.05%)	0 (0.00%)	1 (1.15%)	3 (2.38%)	1 (0.51%)

IQR: Interquartile range; HER2: Human epidermal growth factor receptor 2; PR: Progesterone receptor; NST: No special type.

5.2.4 Luminal A Histological Grade 3 Cases

Supplementary Table 9: Clinical characteristics of patients in the METABRIC cohort with luminal A histological grade 3 breast cancer, classified by CNA Quartiles.

Clinical Variables	Luminal A Grade 3 (N = 172)	Quartile 1 (N = 30)	Quartile 2 (N = 41)	Quartile 3 (N = 59)	Quartile 4 (N = 42)
**Subtype**					
Luminal A	172 (100.00%)	30 (100.00%)	41 (100.00%)	59 (100.00%)	42 (100.00%)
Luminal B	0 (0.00%)	0 (0.00%)	0 (0.00%)	0 (0.00%)	0 (0.00%)
**Histological Grade**					
1	0 (0.00%)	0 (0.00%)	0 (0.00%)	0 (0.00%)	0 (0.00%)
2	0 (0.00%)	0 (0.00%)	0 (0.00%)	0 (0.00%)	0 (0.00%)
3	172 (100.00%)	30 (100.00%)	41 (100.00%)	59 (100.00%)	42 (100.00%)
**Tumour Size**					
mean	27.27	28.97	28.93	25.01	27.62
median (IQR)	25.00 (18.00, 30.00)	22.00 (18.25, 30.00)	25.00 (20.00, 30.00)	22.00 (16.50, 30.00)	26.00 (20.00, 35.00)
**Lymph Nodes Positive**					
mean	2.43	1.44	2.40	2.27	3.33
median (IQR)	0.00 (0.00, 3.00)	0 (0.00, 1.50)	1.00 (0.00, 3.00)	0 (0.00, 2.00)	1.00 (0.00, 4.00)
$\geq$ 1 Lymph node positive	82 (48.81%)	12 (44.44%)	22 (55.00%)	26 (44.07%)	22 (52.38%)
**Age at Diagnosis**					
mean	61.84	64.77	62.20	58.06	64.69
median (IQR)	62.52 (53.29, 70.57)	65.65 (56.50, 76.60)	62.26 (55.65, 69.67)	58.26 (49.30, 69.83)	65.47 (60.08, 69.89)
**HER2 Status**					
Negative	161 (93.60%)	30 (100.00%)	38 (92.68%)	54 (91.53%)	39 (92.86%)
Positive	11 (6.40%)	0 (0.00%)	3 (7.32%)	5 (8.47%)	3 (7.14%)
**PR Status**					
Negative	48 (27.91%)	3 (10.00%)	10 (24.39%)	22 (37.29%)	13 (30.95%)
Positive	124 (72.09%)	27 (90.00%)	31 (75.61%)	37 (62.71%)	29 (69.05%)
**Histological Subtype**					
Ductal/NST	127 (73.84%)	21 (70.00%)	27 (65.85%)	44 (74.58%)	35 (83.33%)
Lobular	17 (9.88%)	2 (6.67%)	7 (17.07%)	7 (11.86%)	1 (2.38%)
Medullary	0 (0.00%)	0 (0.00%)	0 (0.00%)	0 (0.00%)	0 (0.00%)
Mixed	26 (15.12%)	5 (16.67%)	7 (17.07%)	8 (13.56%)	6 (14.29%)
Mucinous	0 (0.00%)	0 (0.00%)	0 (0.00%)	0 (0.00%)	0 (0.00%)
Tubular/Cribriform	0 (0.00%)	0 (0.00%)	0 (0.00%)	0 (0.00%)	0 (0.00%)
Other	1 (0.58%)	1 (3.33%)	0 (0.00%)	0 (0.00%)	0 (0.00%)

IQR: Interquartile range; HER2: Human epidermal growth factor receptor 2; PR: Progesterone receptor; NST: No special type.

6 DSS Multivariable Cox Models

6.1 Final Baseline Multivariable Cox Model

Supplementary Table 10: Final baseline multivariable Cox model for DSS including clinical variables.

Clinical Variable	Beta	SE	HR	95% HR CI	P-value	Significance
PAM50:						
Luminal A (Ref)	-	-	-	-	-	-
Luminal B	0.477	0.123	1.611	(1.266 - 2.050)	<0.001	* * *
Histological Grade:						
1 (Ref)	-	-	-	-	-	-
2	0.477	0.252	1.611	(0.982 - 2.642)	0.059	.
3	0.691	0.258	1.996	(1.204 - 3.310)	0.007	**
Tumour Size	0.014	0.002	1.014	(1.009 - 1.019)	<0.001	* * *
Positive Lymph Nodes	0.049	0.009	1.050	(1.033 - 1.068)	<0.001	* * *
Age at Diagnosis	0.016	0.005	1.017	(1.006 - 1.027)	0.002	**
HER2 Status:						
Negative (Ref)	-	-	-	-	-	-
Positive	0.582	0.200	1.789	(1.208 - 2.649)	0.004	**
Likelihood Ratio Test p-value					<2e-16	* * *
Wald Test p-value					<2e-16	* * *
Score (logrank) Test p-value					<2e-16	* * *

Signifcance codes: 0 (* * *) 0.001 (**) 0.01 (*) 0.05 (.) 0.1()
SE: Standard Error; HR: Hazard Ratio; CI: Confidence Interval

6.2 Final Multivariable Cox Model with CNA Quartiles

Supplementary Table 11: Final multivariable Cox model for DSS including clinical variables and CNA Quartiles.

Clinical Variable	Beta	SE	HR	95% HR CI	P-value	Significance
PAM50:						
Luminal A (Ref)	-	-	-	-	-	-
Luminal B	0.392	0.127	1.480	(1.153 - 1.899)	0.002	**
Histological Grade:						
1 (Ref)	-	-	-	-	-	-
2	0.391	0.254	1.478	(0.899 - 2.432)	0.124	
3	0.525	0.263	1.690	(1.009 - 2.830)	0.046	*
Tumour Size	0.015	0.003	1.015	(1.010 - 1.020)	<0.001	***
Positive Lymph Nodes	0.049	0.008	1.050	(1.033 - 1.068)	<0.001	***
Age at Diagnosis	0.018	0.005	1.018	(1.008 - 1.029)	<0.001	***
HER2 Status:						
Negative (Ref)	-	-	-	-	-	-
Positive	0.514	0.201	1.672	(1.128 - 2.479)	0.011	*
Quartile:						
CNA Q1 (Ref)	-	-	-	-	-	-
CNA Q2	0.040	0.198	1.041	(0.706 - 1.534)	0.840	
CNA Q3	0.504	0.186	1.656	(1.150 - 2.385)	0.007	**
CNA Q4	0.434	0.191	1.544	(1.062 - 2.244)	0.023	*
Likelihood Ratio Test p-value					<2e-16	***
Wald Test p-value					<2e-16	***
Score (logrank) Test p-value					<2e-16	***
Signifcance codes: 0 (***)	0.001 (**)		0.01 (*)	0.05 (.)	0.1()	
SE: Standard Error; HR: Hazard Ratio; CI: Confidence Interval						

6.3 Final Multivariable Cox Model with CNA Quartiles and Interactions

Supplementary Table 12: Final multivariate Cox model for DSS including clinical variables and CNA Quartiles. This model allowed for interactions between histological grade, PAM50 subtype and CNA Quartile.

Clinical Variable	Beta	SE	HR	95% CI	P-value	Significance
PAM50:						
Luminal B (Ref)	-	-	-	-	-	-
Luminal A	-1.069	0.299	0.343	(0.191 - 0.618)	<0.001	***
Histological Grade:						
1 (Ref)	-	-	-	-	-	-
2	0.381	0.254	1.464	(0.889 - 2.410)	0.134	
3	0.528	0.262	1.696	(1.014 - 2.837)	0.044	*
Tumour Size	0.015	0.003	1.015	(1.010 - 1.020)	<0.001	***
Positive Lymph Nodes	0.050	0.008	1.051	(1.034 - 1.069)	<0.001	***
Age at Diagnosis	0.018	0.005	1.018	(1.008 - 1.029)	<0.001	***
HER2 Status:						
Negative (Ref)	-	-	-	-	-	-
Positive	0.541	0.202	1.717	(1.157 - 2.550)	0.007	**
CNA Quartile:						
CNA Q1 (Ref)	-	-	-	-	-	-
CNA Q2	-0.449	0.304	0.638	(0.352 - 1.158)	0.140	
CNA Q3	0.037	0.272	1.038	(0.608 - 1.770)	0.892	
CNA Q4	-0.070	0.258	0.933	(0.563 - 1.546)	0.787	
CNA Quartile by PAM50:						
CNA Q2:LumA	0.764	0.395	2.147	(0.990 - 4.655)	0.053	.
CNA Q3:LumA	0.730	0.364	2.074	(1.017 - 4.231)	0.045	*
CNA Q4:LumA	0.909	0.370	2.482	(1.203 - 5.123)	0.014	*
Likelihood Ratio Test p-value					<2e-16	***
Wald Test p-value					<2e-16	***
Score (logrank) Test p-value					<2e-16	***
Signifcance codes: 0 (***)	0.001 (**)		0.01 (*)	0.05 (.)	0.1()	
SE: Standard Error; HR: Hazard Ratio; CI: Confidence Interval						

7 Cox Proportional Hazards Assumption

Supplementary Table 13: Assessing the proportional hazards assumption of the final multivariable Cox model including selected clinical variables and CNA Quartiles, allowing for interactions. Carried out using scaled Schoenfeld residuals with time, to test for independence between residuals and time.

Clinical Variable	Chisq	DF	P-value
PAM50 Subtype	3.611	1	0.05741
Histological Grade	1.901	2	0.38660
Tumour Size	0.378	1	0.53858
Positive Lymph Nodes	0.225	1	0.63511
Age at Diagnosis	2.263	1	0.13250
HER2 Status	2.410	1	0.12053
CNA Quartile	12.859	3	0.00495
CNA Quartile:PAM50	16.385	3	0.00095
Global	28.221	13	0.00843
Chisq: Chi-squared test; DF: Degrees of freedom.			

8 Recursive Partitioning Survival Trees

8.1 Survival Trees with CNA Quartile

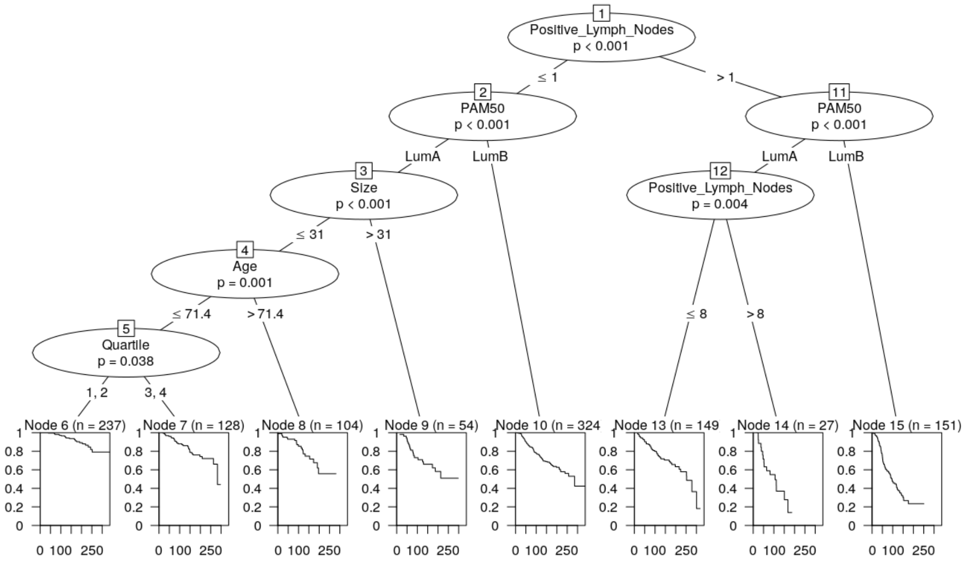

Supplementary Figure 1: Recursive partitioning survival tree considering the six significant clinical variables and CNA Quartiles.

9 Tishchenko et al Material

9.1 Tishchenko Up-Regulated Genes

Supplementary Table 14: List of top ten most up-regulated genes in the Tishchenko et al [20] study.

Gene Symbol	Gene Name
POLQ	DNA Polymerase Theta
CKAP2L	Cytoskeleton Associated Protein 2 Like
KIFC1	Kinesin Family Member C1
FOXM1	Forkhead Box M1
TROAP	Trophinin Associated Protein
UBE2C	Ubiquitin Conjugating Enzyme E2C
AURKB	Aurora Kinase B
NCAPG	Non-SMC Condensin I Complex Subunit G
HJURP	Holliday Junction Recognition Protein
MCM10	Minichromosome Maintenance 10 Replication Initiation Factor

9.2 Tishchenko Quantiles across CNA Quartiles

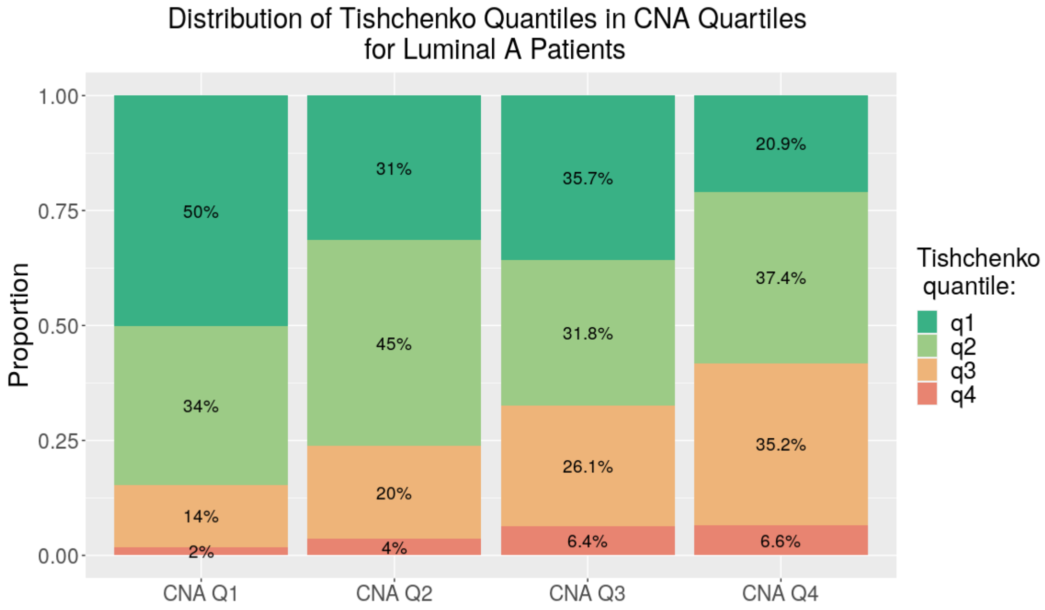

Supplementary Figure 2: Stacked barplot showing the proportion and percentage of patients assigned to each Tishchenko quantile within each CNA Quartile in luminal A patients.

10 Additional Information

10.1 Contingency Table by PAM50, Grade and CNA Quartile

Supplementary Table 15: Three-way contingency table containing PAM50, histological grade and CNA Quartile. Note that information about histological grade was missing for 50 patients.

		**Grade**			**Total**
**PAM50**	**Quartile**	**1**	**2**	**3**	**-**
**Luminal A**	**1**	54	129	30	213
	**2**	38	119	41	198
	**3**	19	85	59	163
	**4**	7	45	42	94
**Luminal B**	**1**	7	34	22	63
	**2**	5	49	29	83
	**3**	3	53	66	122
	**4**	3	50	136	189
**Total**	**-**	136	564	425	1125

References

[1] T. Nielsen, B. Wallden, C. Schaper, S. Ferree, S. Liu, D. Gao, G. Barry, N. Dowidar, M. Maysuria, and J. Storhoff. Analytical validation of the PAM50-based Prosigna Breast Cancer Prognostic Gene Signature Assay and nCounter Analysis System using formalin-fixed paraffin-embedded breast tumor specimens. *BMC Cancer*, 14:177, 2014.

[2] S. K. Chia, V. H. Bramwell, D. Tu, L. E. Shepherd, S. Jiang, T. Vickery, E. Mardis, S. Leung, K. Ung, K. I. Pritchard, J. S. Parker, P. S. Bernard, C. M. Perou, M. J. Ellis, and T. O. Nielsen. A 50-gene intrinsic subtype classifier for prognosis and prediction of benefit from adjuvant tamoxifen. *Clin. Cancer Res.*, 18(16):4465–4472, Aug 2012.

[3] S. J. Dawson, O. M. Rueda, S. Aparicio, and C. Caldas. A new genome-driven integrated classification of breast cancer and its implications. *EMBO J.*, 32:617–628, 2013.

[4] E. A. Rakha, J. S. Reis-Filho, F. Baehner, D. J. Dabbs, T. Decker, V. Eusebi, S. B. Fox, S. Ichihara, J. Jacquemier, S. R. Lakhani, J. Palacios, A. L. Richardson, S. J. Schnitt, F. C. Schmitt, P. H. Tan, G. M. Tse, S. Badve, and I. O. Ellis. Breast cancer prognostic classification in the molecular era: the role of histological grade. *Breast Cancer Res.*, 12(4):207, 2010.

[5] Mahul B Amin, Stephen B Edge, Frederick L Greene, David R Byrd, Robert K Brookland, Mary Kay Washington, Jeffrey E Gershenwald, Carolyn C Compton, Kenneth R Hess, Daniel C Sullivan, J Milburn Jessup, James D Brierley, Lauri E Gaspar, Richard L Schilsky, Charles M Balch, David P Winchester, Elliot A Asare, Martin Madera, Donna M Gress, and Laura R Meyer. *Principles of Cancer Staging.* Springer, New York ;, 7th ed. / edition, 2017.

[6] F. Tonellotto, A. Bergmann, K. de Souza Abrahao, S. S. de Aguiar, M. A. Bello, and L. C. S. Thuler. Impact of Number of Positive Lymph Nodes and Lymph Node Ratio on Survival of Women with Node-Positive Breast Cancer. *Eur J Breast Health*, 15(2):76–84, Apr 2019.

[7] Y. Fong, J. Evans, D. Brook, J. Kenkre, P. Jarvis, and K. Gower-Thomas. The Nottingham Prognostic Index: five- and ten-year data for all-cause survival within a screened population. *Ann R Coll Surg Engl*, 97:137–139, 2015.

[8] Banyameen. Iqbal and Archana. Buch. Hormone receptor (ER, PR, HER2/neu) status and proliferation index marker (Ki-67) in breast cancers: Their onco-pathological correlation, shortcomings and future trends. *Medical Journal of Dr. D.Y. Patil University*, 9:674–679, 2016.

[9] C. Curtis, S. P. Shah, S. F. Chin, G. Turashvili, O. M. Rueda, M. J. Dunning, D. Speed, A. G. Lynch, S. Samarajiwa, Y. Yuan, S. Graf, G. Ha, G. Haffari, A. Bashashati, R. Russell, S. McKinney, A. Langerod, A. Green, E. Provenzano, G. Wishart, S. Pinder, P. Watson, F. Markowetz, L. Murphy, I. Ellis, A. Purushotham, A. L. Borresen-Dale, J. D. Brenton, S. Tavare, C. Caldas, S. Aparicio, C. Caldas, S. Aparicio, C. Curtis, S. P. Shah, C. Caldas, S. Aparicio, J. D. Brenton, I. Ellis, D. Huntsman, S. Pinder, A. Purushotham, L. Murphy, C. Caldas, S. Aparicio, C. Caldas, H. Bardwell, S. F. Chin, C. Curtis, Z. Ding, S. Graf, L. Jones, B. Liu, A. G. Lynch, I. Papatheodorou, S. J. Sammut, G. Wishart, S. Aparicio, S. , K. Gelmon, D. Huntsman, S. McKinney, C. Speers, G. Turashvili, P. Watson, I. Ellis, R. Blamey, A. Green, D. Macmillan, E. Rakha, A. Purushotham, C. Gillett, A. Grigoriadis, S. Pinder, E. de Rinaldis, A. Tutt, L. Murphy, M. Parisien, S. Troup, C. Caldas, S. F. Chin, D. Chan, C. Fielding, A. T. Maia, S. McGuire, M. Osborne, S. M. Sayalero, I. Spiteri, J. Hadfield, S. Aparicio, G. Turashvili, L. Bell, K. Chow, N. Gale, D. Huntsman, M. Kovalik, Y. Ng, L. Prentice, C. Caldas, S. Tavare, C. Curtis, M. J. Dunning, S. Graf, A. G. Lynch, O. M. Rueda, R. Russell, S. Samarajiwa, D. Speed, F. Markowetz, Y. Yuan, J. D. Brenton, S. Aparicio, S. P. Shah, A. Bashashati, G. Ha, G. Haffari, and S. McKinney. The genomic and transcriptomic architecture of 2,000 breast tumours reveals novel subgroups. *Nature*, 486:346–352, 2012.

[10] Ghina B. Fakhri, Reem S. Akel, Maya K. Khalil, Deborah A. Mukherji, Fouad I. Boulos, and Arafat H. Tfayli. Concordance between immunohistochemistry and microarray gene expression profiling for estrogen receptor, progesterone receptor, and her2 receptor statuses in breast cancer patients in lebanon. *International Journal of Breast Cancer*, 2018:1–6, 2018.

[11] T. V. Hansen, J. Vikesaa, S. S. Buhl, H. H. Rossing, V. Timmermans-Wielenga, and F. C. Nielsen. High-density SNP arrays improve detection of HER2 amplification and polyploidy in breast tumors. *BMC Cancer*, 15:35, 2015.

[12] Sally Agersborg, Christopher Mixon, Thanh Nguyen, Sramila Aithal, Sucha Sudarsanam, Forrest Blocker, Lawrence Weiss, Robert Gasparini, Shiping Jiang, and Wayne et al. Chen. Immunohistochemistry and alternative fish testing in breast cancer with her2 equivocal amplification. *Breast Cancer Research and Treatment*, 170(2):321–328, 2018.

[13] M. H. Amer. Genetic factors and breast cancer laterality. *Cancer Manag Res*, 6:191–203, 2014.

[14] N. L. Henry and L. A. Cannon-Albright. Breast cancer histologic subtypes show excess familial clustering. *Cancer*, 2019.

[15] G. N. Sharma, R. Dave, J. Sanadya, P. Sharma, and K. K. Sharma. Various types and management of breast cancer: an overview. *J Adv Pharm Technol Res*, 1(2):109–126, Apr 2010.

[16] P. Nygren. What is cancer chemotherapy? *Acta Oncol*, 40(2-3):166–174, 2001.

[17] R. Baskar, K. A. Lee, R. Yeo, and K. W. Yeoh. Cancer and radiation therapy: current advances and future directions. *Int J Med Sci*, 9(3):193–199, 2012.

[18] A. Awan and K. Esfahani. Endocrine therapy for breast cancer in the primary care setting. *Curr Oncol*, 25(4):285–291, 08 2018.

[19] F. McCrate, E. Dicks, E. Powell, J. Chafe, R. Roome, C. Simmonds, and H. Etchegary. Surgical treatment choices for breast cancer in Newfoundland and Labrador: a retrospective cohort study. *Can J Surg*, 61(6):377–384, 12 2018.

[20] I. Tishchenko, H. H. Milioli, C. Riveros, and P. Moscato. Extensive Transcriptomic and Genomic Analysis Provides New Insights about Luminal Breast Cancers. *PLoS ONE*, 11:e0158259, 2016.
